# Actor Perceptions of the Governance Framework and Non-Carbon Benefits from the Ghana Cocoa Forest REDD+ Program: An Extended Q-Study of the Juabuso-Bia Hotspot Intervention Area

**DOI:** 10.1007/s00267-024-01978-2

**Published:** 2024-04-30

**Authors:** Frank Akowuge Dugasseh, Marshall Alhassan Adams, Marianne Zandersen

**Affiliations:** 1 Department of Environment Science and iClimate Interdisciplinary Center for Climate Change, Aarhus University, Aarhus, Denmark; 2 United States Forest Service International Programs, Africa Regional Environmental, Programs Office, Accra, Ghana

**Keywords:** REDD+, Non-carbon benefits, Hotspot intervention area, Cocoa, Q-methodology, Ghana Cocoa Forest REDD+ program

## Abstract

The expansion of cocoa farms is a major driver of deforestation and emissions in Ghana’s high forest zone. The Ghana Cocoa Forest Reducing Emissions from Deforestation and Forest Degradation Program (REDD+) was launched as the world’s first commodity-based initiative to address emissions from deforestation caused by cocoa production and generate non-carbon benefits. Hotspot Intervention Areas were established to implement the Ghana Cocoa REDD+ program. This study combines Q-methodology with focus group discussions and interviews to assess stakeholder perceptions in the Juabuso-Bia cocoa landscape regarding the capacity of the Hotspot Intervention Area to facilitate the generation of governance and economic non-carbon benefits to sustain emission reductions. We found that introducing the Hotspot Intervention Area has re-centralized landscape governance, which, coupled with weak collaboration among stakeholders, has led to poor generation of non-carbon benefits. Furthermore, efforts to include women in the leadership structure of the Hotspot Intervention Area can be described as tokenism, and little has been done to improve land and tree tenure for vulnerable groups. This, combined with the low adoption of climate-smart cocoa practices, is likely to negatively affect the generation of economic non-carbon benefits. To overcome these challenges, we recommend reforming the Hotspot Intervention Area, bolstering community-level sensitization, improving access to decision-making spaces that will enhance the participation of women and minority groups in landscape governance, and improving farmers’ tenure security through a registration scheme for land and trees. These recommendations can ensure the efficient generation of non-carbon benefits, which are key to the success of REDD+.

## Introduction

The last two decades have seen REDD+ become the most used mechanism for reducing forest loss and emissions in the tropics through a performance-based reward scheme. REDD+ is a program under the United Nations Framework Convention on Climate Change (UNFCCC) and stands for Reduced Emissions from Deforestation and Forest Degradation, with the ‘+’ signifying the role of conservation, sustainable management of forests, and enhancement of carbon stocks (Andoh and Lee 2018; Hugel et al. 2018; UNFCCC 2014). About 56 countries have included REDD+ programs in their Nationally Determined Contributions as mechanisms for addressing climate change (Maniatis et al. 2021). Communities in and around tropical forests rely on forest resources for a significant part of their livelihoods (Angelsen et al. 2014; FAO 2018; Schmid 2022). Therefore, REDD+ implementation needs to provide mechanisms to generate and share benefits equitably, in addition to achieving effective reductions in emissions (Dunlop and Corbera 2016; Soliev et al. 2021). Recognizing this, REDD+ programs have evolved to include non-carbon benefits (NCBs) to reduce the negative incentives that drive deforestation and forest degradation and support the long-term sustainability of emission reductions (Hvalkof and Krøijer 2013; Katerere et al. 2015). NCBs encompass social, cultural, and economic safeguards (e.g., improve livelihoods, sustain local knowledge), governance (e.g., create secure tenure regimes, promote inclusion and participation of marginalized groups), and environmental improvements (e.g., watershed management and biodiversity conservation) (Duchelle et al. 2018; Katerere et al. 2015).

Related to governance and livelihoods, NCBs have been shown to improve indigenous peoples’ voice in forest governance in Costa Rica (Wallbott and Florian-Rivero 2018), increase the value of alternative income sources in Sierra Leone (Malan et al. 2024), and improve environmental conditions by reducing tree-cover loss in Guyana, at least during the program period (Roopsind et al. 2019). NCBs are also commonly termed ‘co-benefits’, ‘multiple’, or ‘ancillary’ benefits (Hugel et al. 2018; Karlsson et al. 2020; Mayrhofer and Gupta 2016; Uisso et al. 2021). This paper uses NCBs to include all such terms.

In this paper, we focus on the Ghana Cocoa-Forest REDD+ Program (Ghana Cocoa REDD+). The program aims to reduce deforestation and the degradation of forests resulting from cocoa production. Ghana launched the Ghana Cocoa REDD+ in 2019 to curb cocoa-induced deforestation and generate NCBs for the farmers involved. It created the Hotspot Intervention Area (HIA) governance framework, a community-based governance model and associated instruments aimed at avoiding governance paralysis, which has been observed to result from top-down approaches in some REDD+ initiatives (Gupta et al. 2016; Makatta et al. 2015; Nuesiri 2017; Rodriguez-Ward et al. 2018).

Traditionally, REDD+ programs are often implemented in landscapes with some level of protection, where livelihoods typically depend on non-timber forest products (Malan et al. 2024; Parrotta et al. 2022; Wunder et al. 2020). However, Ghana Cocoa REDD+ differs by being the first program to focus on a commodity value chain with diverse actors, authorities, and interests. Considering its novelty and recognizing the importance of NCBs in REDD+ implementation, this paper examines the extent to which the various actors see the HIA as a viable governance framework for implementing REDD+. In particular, it investigates the HIA’s effectiveness in delivering the governance and economic NCBs that are highly prioritized by the Forestry Commission (Forestry Commission 2017, 2020, 2021).

The Ghana Cocoa REDD+ also provides a test case to evaluate the governance instruments, such as strategies, policies, and quasi-legislation^1^, designed to provide direction and grounding for the Ghana Cocoa REDD+ (Forestry Commission 2017; NCRC 2020).

In assessing actor perceptions of Ghana Cocoa REDD+ NCBs, we pay particular attention to participation by women and vulnerable groups in landscape^2^ governance and share of NCBs, as these women continue to be beyond the influence of REDD+. This is because REDD+ risks exacerbating gender inequalities and restricting women’s access to decision-making and benefit-distribution processes (Arwidaa et al. 2017), and the well-being of women appears to be poor compared to other groups in communities where REDD+ is implemented (Larson et al. 2018). Moreover, women are the least equipped to face the risks and impacts of deforestation. At the same time, there are substantial obstacles to their engagement in REDD+ initiatives because of their insecurity of tenure and inequitable rights (Bashiru 2022; Soliev et al. 2021).

Against this background, we address the following research question: How do actors perceive the practices and capacity of the HIA to deliver i) governance NCBs as applied to landscape governance, inclusion and participation, land, and tree tenure; and ii) economic and social NCBs in relation to livelihood improvement and poverty reduction?

We apply Q-methodology (see the Methods and Materials section for an explanation) to identify different perspectives and narratives of the Ghana Cocoa REDD+ and to guide the analysis. Interviews with different stakeholders in the cocoa sector enrich and contextualize perspectives for a deeper understanding of issues at play, while focus group discussions allow for in-depth discussions and sharing of thoughts and perceptions, providing more nuanced and natural feedback.

The next section describes the conceptual background to the linkages between incentives and REDD+ NCBs. It also provides contextual background on the Ghana Cocoa REDD+ and the governance arrangements in which it is embedded. Next, we present the methods and materials used in this study. After presenting the findings, we discuss their broader implications and conclude with suggestions for further research and recommendations.

## Conceptual and Contextual Background

### Linkages between Incentives and REDD+ Non-Carbon Benefits

In the context of REDD+, incentives comprise collections of targeted actions, investments, policy instruments, or direct benefits meant to influence the behavior of forest communities toward sustainable land-use practices (Cheney et al. 2017; Khaine et al. 2019). REDD+ incentives can be result-based, such as payments for emission reductions from deforestation and forest degradation, or non-result-based, such as the generation of NCBs in some REDD+ programs (Brockhaus et al. 2017; Karsenty and Ongolo 2012; Katerere et al. 2015). However, the ability of incentives to deliver emission reduction outcomes or improve the livelihoods of forest communities lies with the potential trade-offs that beneficiaries would have to make regarding the expected generation of value (Cheney et al. 2017; Visseren-Hamakers et al. 2012).

Incentives are pivotal in REDD+ design, and one such collection of incentives is characterized as NCBs. In the early stages of REDD+, incentives were mainly focused on payments for emission reductions, with less attention given to NCBs (Duchelle et al. 2018; Wunder et al. 2020). However, when REDD+ pilots demonstrated that the programs needed enabling conditions (e.g., policy reforms) and additional benefits (e.g., livelihood improvements) to sustain emission reductions, NCBs were included in subsequent REDD+ programs (Duchelle et al. 2018; Hailemariam et al. 2015; Uisso et al. 2021). Civil society organizations and indigenous communities were among the first to advocate for the inclusion of NCBs. NCBs were included in the 2010 Cancun Agreements (Decision 1/ CP.16) and reiterated in later agreements (see Table 1).

**Table 1:** The Trajectory from RED to REDD+ and the Inclusion of Non-Carbon Benefits

Conference of the Parties	Carbon Benefits	Non-Carbon Benefits
Kyoto COP3 (1997)	**RED -** Reducing Emissions from Deforestation.	Nil
Bali COP13 (2007)	**REDD** - Reducing Emissions from Deforestation and Forest Degradation.	Nil
Poznan COP14 (2008); Copenhagen COP15 (2009); Cancún COP16 (2010); Warsaw COP19 (2013); Paris COP21 (2015)	**REDD**+ Reducing Emissions from Deforestation and Forest Degradation, including the role of conservation, sustainable management of forests, and enhancement of forest carbon stocks in developing countries.	Social, environmental, and governance benefits.

REDD+ program initiators and funders such as UN-REDD, the World Bank, and the voluntary carbon market players use various approaches to incentivize NCBs. Katerere et al. (2015) identified four such incentivizing in REDD+ programs: composite, eligibility, premium, and unbundled

The NCBs aim to enhance social, economic, and environmental benefits, incentivize ecosystem conservation, and promote effective forest governance (Katerere et al. 2015). To influence land-use practices that lead to reduced emissions, NCBs need to motivate and compensate forest communities through incentives (Cheney et al. 2017; Duchelle et al. 2018; Visseren-Hamakers et al. 2012). These are among the reasons why the Cancún Safeguards recommended the generation of NCBs in emission reduction programs (Hugel et al. 2018; Katerere et al. 2015; Uisso et al. 2021).

In the composite approach, NCBs are seamlessly incorporated into the conceptualization, design, and execution stages of REDD+ programs, and NCB outcomes are therefore assessed before payment. The composite approach prioritizes grassroots involvement and aligns with the mandates of UNFCCC and the Green Climate Fund. A typical example is the Floresta REDD+ Pilot Program funded by the Green Climate Fund in Brazil.

Under the eligibility approach, countries demonstrate that they have incorporated NCBs into their REDD+ programs before they are eligible for REDD+ funding. This approach is favored by multinational institutions such as the World Bank and is the model underlying the Ghana Cocoa REDD+.

The premium approach rewards emission reduction programs that also generate NCBs and is a market-based mechanism associated with the voluntary carbon market. This approach is based on the premise that investors are increasingly becoming more interested in emissions. reductions that also generate social, environmental, and governance benefits supporting sustainable development. The Kasigau Corridor REDD+ project in Kenya is an example.

The non-bundled approach explores separate additional mechanisms to incentivize or pay for NCBs generated within a REDD+ emission reduction program. This approach is common in Latin American countries, with an example being the Socio Bosque program in Ecuador.

Table 2 provides an overview of the different types of NCBs described in the literature and their contribution to achieving emission reductions. The listed NCBs can be incentivized following the different approaches described above. NCBs, as integral components of REDD+ safeguards, play a crucial role in mitigating potential risks and negative consequences associated with REDD+ programs (Hvalkof and Krøijer 2013; Katerere et al. 2015). NCBs, therefore, can make a wide range of contributions to social, cultural, economic, governance, and environmental aspects of REDD+. Due to this broad range, NCBs can facilitate close collaborations between REDD+ programs and local communities but can also affect participation when incentives are inadequate (Awung and Marchant 2020). The Ghana Cocoa REDD+ introduced in the next subsection illustrates such a broad partnership.

**Table 2:** Types of Non-Carbon Benefits

Type	Non-carbon benefits contribution to REDD+	References
Social, cultural, economic	• Maintain sustainable livelihoods, cultures, and communities.	3, 1, 2, 5
	• Enhance and support the social value of forests.	
	• Sustain cultural services and traditional knowledge.	
	• Enhance the security of populations in forest landscapes.	
	• Facilitate the empowerment of individuals and communities.	
	• Create opportunities for wealth creation and improved well-being.	
	• Support indigenous and community-conserved areas for cultural spiritual and services.	
	• Enhance food security and dynamic subsistence forest economies.	
	• Support income generation and employment opportunities.	
Governance	• Strengthen local/traditional decision-making processes.	3, 4, 1, 2, 5
	• Promote accountability, equity, participation, and transparency in forest management.	
	• Strengthen multi-stakeholder consultation and dialogue on the forest.	
	• Strengthen sub-national governance structures and institutions.	
	• Address land tenure to improve rights and reduce conflicts.	
	• Streamline land and carbon rights for inclusive incentive allocation.	
	• Support the design and implementation of equitable distribution of benefits.	
	• Strengthen legal frameworks, compliance, and law enforcement.	
	• Support community participation in policies that improve the management of forest resources.	
	• Operationalize safeguards information systems and grievance redress mechanisms.	
	• Support gender mainstreaming in forest governance.	
Environment	• Conserve and protect biodiversity to increase ecosystem resilience.	4, 3, 1, 2, 5
	• Protect and maintain ecosystem services.	
	• Monitor biodiversity at the national and community levels.	
	• Secure medicinal plants and enhance curative practices.	
	• Improve water regulation and watershed management.	

1 = Uisso et al. (2021), 2 = Duchelle et al. (2018), 3 = Katerere et al. (2015), 4 = Hailemariam et al. (2015), 5 = Hvalkof and Krøijer (2013)

### Ghana Cocoa REDD+ and its Governance Framework

Ghana is a leading producer of cocoa, with the commodity representing the backbone of the country’s economy due to the contribution it makes to employment and foreign exchange generation (Adams and Carodenuto 2023; Gneiting and Arhin 2023). An estimated 800,000 smallholder farmers derive their livelihood from cocoa farming (Nasser et al. 2020). The high demand for cocoa beans from the confectionery industry contributes to the expansion of cocoa farms into protected areas in the high forest zone, causing forest losses (Julian et al. 2022; van der Haar et al. 2023). The high forest zone is the forested eco-region of southern Ghana, which has significant biodiversity and is part of the Guinean forests of West Africa (Hawthorne 1995), covering approximately 82,000 km^2^ (Oduro 2016).

With an annual deforestation rate of 3.6% (Forestry Commission 2021), Ghana is experiencing one of the highest levels of deforestation in the world (Nyarko et al. 2023). Smallholder farmers are major contributors to forest cover loss in the high forest zone (Forestry Commission 2017, 2021). Agricultural expansion into forests accounts yearly for losses of more than 130,000 hectares of the high forest zone, and one-third of this deforestation is attributed to cocoa production (Forestry Commission 2017). This makes cocoa an important driver of deforestation in the high forest zone.

The current deforestation trend could negatively impact cocoa production over time by deteriorating microclimatic conditions that support cocoa production (Ruf et al. 2015). To tackle these risks, the Forestry Commission of Ghana, and the Ghana Cocoa Board (COCOBOD)^3^ initiated the Ghana Cocoa REDD+ in 2019. The program is included in Ghana’s Nationally Determined Contribution to the Paris Agreement (EPA 2021) and aims to reduce carbon emissions from cocoa-induced deforestation and improve productivity on existing cocoa farms through climate-smart cocoa practices. An emission reduction payment agreement was signed between the Government of Ghana and the World Bank in 2019 that rewards community efforts to reduce carbon emissions from deforestation and forest degradation (Forestry Commission 2021).

Implementation costs of the Ghana Cocoa REDD+ include an estimated $230 million for the first five years, with funding anticipated from emission reduction payments (21%), the government of Ghana (23%), the cocoa industry (51%), and donor grants (5%) (Forestry Commission 2021). The projected emission reductions are anticipated to reach 10 million tons of carbon dioxide equivalent (tCO_2_e) in the first phase (2019–2025) (Forestry Commission 2017). The World Bank’s Forest Carbon Partnership Facility Fund anticipates a performance-based payment to Ghana of a maximum of US$50 million if this first phase is successful (Forestry Commission 2020).

Ghana Cocoa REDD+ focuses on specific high deforestation hotspots, supporting farmers to increase cocoa production without encroaching on protected forest areas. Through NCBs, the program aims to help secure predictable income streams for farmers through cocoa intensification, agroforestry, and increased premium payments. The Ghana Cocoa REDD+ supports climate-smart cocoa farming practices that are expected to increase cocoa production per hectare on existing cocoa plots, which is assumed to lead to reduced encroachment on forest lands. The program expects to double annual cocoa yields from 400 kg/ha/yr to 800 kg/ha/yr, leading to an additional economic value per Hotspot Intervention Area (HIA) of close to USD 16 million for about 24,000 cocoa farmers, amounting to USD 676 per year per farmer – a key economic NCB (Forestry Commission 2020). Table 3 presents a list of NCBs that the Ghana Cocoa REDD+ is expected to generate through the HIA governance framework. The program intends to incentivize the listed NCBs following the eligibility approach described earlier.

**Table 3:** NCBs in Ghana Cocoa REDD+

Landscape stakeholder beneficiaries of NCBs	Incentive type	NCB description	Responsibility for delivering the NCB	Priority level	Main NCB categories
Registered farmers under Ghana Cocoa REDD+	Non-monetary	Access to cocoa extension and agronomic resources.	Government	High	Economic (livelihood)
	Non-monetary	Distribution of farm inputs and planting materials.	Government	High	Economic (livelihood)
	Non-monetary	Establishment of nurseries to supply cocoa seedlings.	Government	High	Economic (livelihood)
	Monetary	Increased income through the sale of certified cocoa beans.	Government, private sector.	High	Economic (livelihood)
	Monetary	Increase in cocoa yields per hectare.	Government, private sector.	High	Economic (livelihood)
	Monetary	Rehabilitation of diseased and unproductive cocoa farms.	Government	High	Economic (livelihood)
	Monetary	Additional and diversification of livelihood options.	Government, NGOs, private sector.	High	Economic (livelihood)
	Monetary	Financial inclusion through VSLA, fintech, and supply of inputs or financial credit.	Government, NGOs, private sector.	High	Economic (livelihood)
	Non-monetary	Tree and carbon rights identification and registration.	Government, traditional council.	High	Governance
	Non-monetary	Land tenure security improvement.	Government, traditional council,	High	Governance
	Non-monetary	Training and capacity building in CSC.	Government.	High	Governance
	Non-monetary	Mapping and registration of cocoa farms.	Government, private sector	High	Governance
Traditional authorities	Non-monetary	Landscape governance and management improvement	Traditional council, government, HIA.	High	Governance
	Non-monetary	Law enforcement and compliance improved.	Traditional council, government, HIA.	High	Governance
HIA/CREMA communities	Non-monetary	Landscape governance and management improvement	Government, NGOs	High	Governance
	Non-monetary	Infrastructural development using premium payments	Private sector, NGOs	High	Economic (livelihood)
	Non-monetary	Certification of cocoa-related activities	Private sector, NGOs	High	Economic /Ecosystem
Forestry Commission	Non-monetary	Law enforcement and compliance	HIAs, traditional council, Government.	High	Governance
	Non-monetary	Collaborative forest management	HIAs, traditional council, Government.	High	Governance
	Non-monetary	Creation of a public-private forest conservation/rehabilitation fund.	Government, private sector, NGOs.	High	Governance
Ghana Cocoa Board	Monetary	Increase cocoa production	Ghana Cocoa Board	Medium	Economic (livelihood)
	Monetary	Sale of climate smart cocoa beans at a premium price	HIA	Medium	Economic (livelihood)
	Non-monetary	Development of cocoa management systems	Government	Medium	Governance
Municipal and district assemblies	Non-monetary	Landscape governance and management improved.	Traditional authorities, private sector, NGOs.	High	Governance
Private sector	Non-monetary	Cultivation and purchase of climate-smart cocoa beans.	Private sector, government, NGOs.	High	Economic (livelihood)
		Supply chain efficiency from HIA to the port for export	Private sector, government, NGOs.	Low	Governance
		Implementation of cocoa traceability systems	Private sector, government, NGOs.	Low	Governance

Source: Adapted from Forestry Commission (2021; 2020; 2017)

Village Savings and Loans Associations

Climate-smart cocoa

The HIA framework was designed as part of the implementation strategy for Ghana Cocoa REDD+. The HIA is a landscape governance framework that evolved from the Community Resource Management Area (CREMA) framework, which integrates wildlife conservation, land-use practices, and rural development to benefit resource-rich communities (Asare et al. 2013; Bayala 2024, this issue; Foli et al. 2018). CREMA evolved initially from wildlife conservation to improve wildlife connectivity and support sustainable resource use outside protected areas (Damnyag et al. 2013). However, a challenge for existing CREMAs has been their relatively small size (typically 25,000–30,000 ha) and the difficulty of ensuring sufficient wildlife connectivity within them. Therefore, the HIA was created to cover a larger area (more than 100,000 ha) to facilitate connectivity and sufficient potential for emission reduction (NCRC 2020).

The HIA governance framework aims to address perverse incentives that drive unsustainable land-use practices by reconnecting land ownership with forest governance (Mason et al. 2016). The objective of the HIA governance framework is to initiate landscape interventions (e.g., the development of land-use plans) through a bottom-up approach and facilitate a consultative process that reflects the priorities, values, and customs of forest communities (van der Haar et al. 2023). Another objective of the HIA is to improve the participation of forest communities and minority groups in landscape governance. This is achieved by linking community stakeholders (farmers, communities, landowners, and traditional leaders) to a consortium of partners (cocoa companies, government, and civil society actors) involved in the cocoa value chain (Forestry Commission 2021; NCRC 2020). The Ghana Cocoa REDD+ is currently being implemented in six HIAs – Juabuso-Bia, Kakum, Atewa, Asuiti-Asunafo, Sefwi-Wiaso-Biabiani, and Ahafo-Ano, all within the high forest zone.

This study focuses on the Juabuso-Bia HIA, which is the most advanced HIA under the Ghana Cocoa REDD+. Administratively, it comprises a 12-member HIA Management Board, heading the HIA, 6 sub-HIA Executive Committees, and 10 CREMAs. The committee members at all levels are elected, but there is also a quota system in place to ensure that women are represented at all levels of the HIA, ensuring their voices are heard and their interests are represented in decision-making. Members of the HIA Management Board are elected from the sub-HIAs. There are also representatives of consortium members (including the Ghana Forestry Commission and Cocoa Board, private sector companies, and civil society organizations) who provide technical support to the HIA Management Board but have no voting rights.

**Fig. 1:**
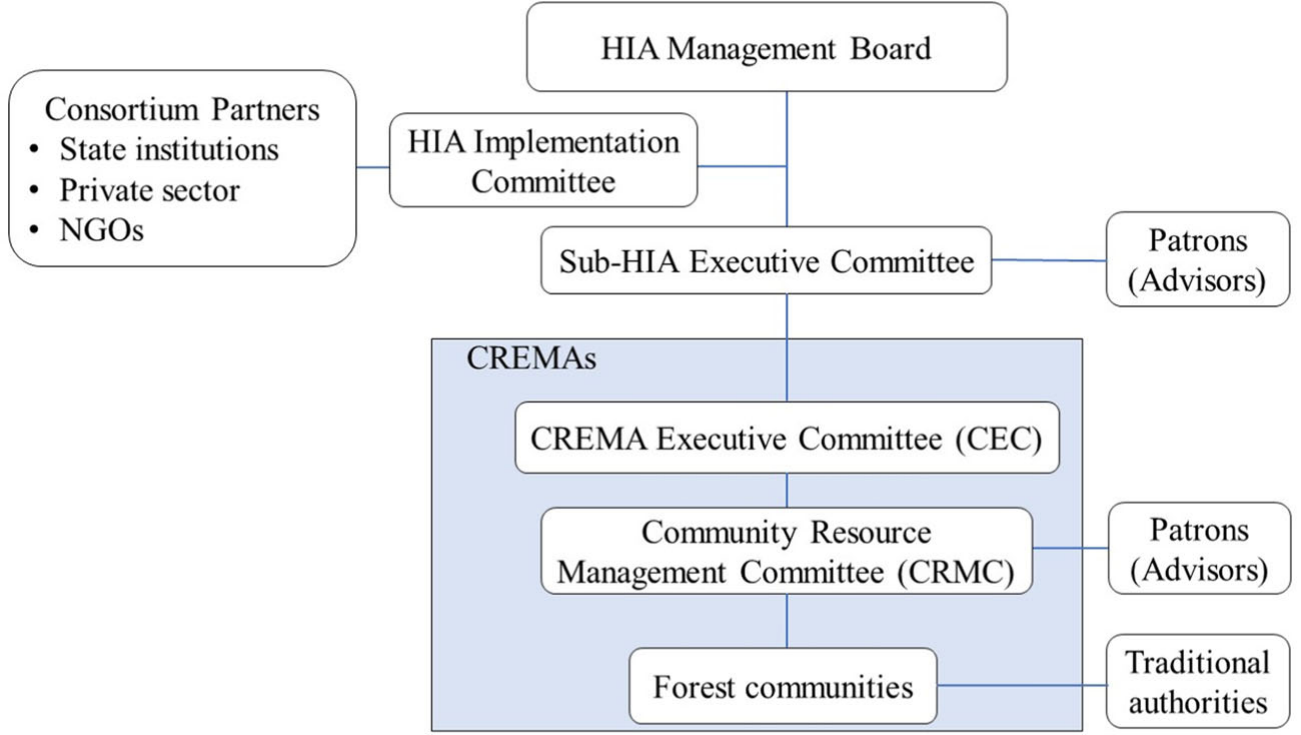
Organigram of the HIA

The HIA Management Board is responsible for providing direction for natural resource management for the benefit of farmers and the ecosystems within the landscape. The HIA Implementation Committee provides a crucial link in facilitating collaboration between the HIA Management Board and consortium partners. A patron, who is usually a traditional leader, is appointed to each of the sub-HIA Executive Committees and to each of the Community Resources Management Committees to offer advisory support. Under the HIA Management Board, sub-HIA Executive Committees oversee the sub-HIA administration and are responsible for developing management plans to define land use practices (van der Haar et al. 2023). These sub-HIA Executive Committees are elected from the CREMA Executive Committees. Part of the responsibilities of the CREMA Executive Committees is to enforce by-laws and conduct forest monitoring activities. The CREMA Executive Committee comprises representatives of the Community Resource Management Committees, who are responsible for implementing CREMA activities at the community level. Each CREMA consists of at least two communities (NCRC 2020). Figure 1 provides an overview of the organizational structure of the HIA.

## Methods and materials

### Study area

**Fig. 2:**
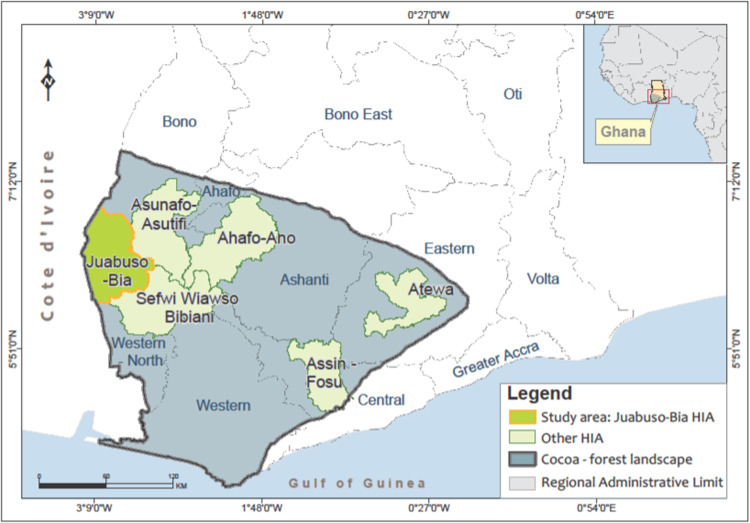
**Map of the Study Area**. **Source**: Authors’ own construction using data from Ghana Data Initiative, Ghana Statistical Service and Natural Earth

The Juabuso-Bia HIA is located in the Western North Region of Ghana and covers an area of 243,561 hectares (Adjei 2021). The landscape is part of the last fragments of the Guinean Forests of the West Africa Biodiversity Hotspot (Carr et al. 2015) and includes protected areas such as the Bia National Park and Krokosua Forest Reserve (Proforest 2021). The Juabuso-Bia HIA encompasses the Juabuso and Bia West Districts and has a total population of about 295,000, with most of the population engaged in cocoa and oil palm cultivation (GSS 2021; IDH and Touton 2018). Before REDD+ interventions, the landscape benefited from cocoa-forest interventions by consortium partners to promote climate-smart cocoa aimed at increasing cocoa production and improving landscape governance (Nasser et al. 2020). It was within the implementation of these interventions that the existing CREMA communities were established. Figure 2 shows the map of the study area.

We selected the Juabuso-Bia HIA as it was the first HIA to be established and has the most advanced governance structure of all the HIAs in Ghana. The cocoa landscape in the Juabuso-Bia HIA is the most productive in Ghana, producing approximately 60,000 MT of cocoa (IDH and Touton 2018) with the major cocoa buying companies operating in the landscape under license from COCOBOD to buy cocoa from farmers and sell it to the Board. The landscape is also important for the production of major forest-risk export commodities like oil palm (Proforest 2021). Consequently, due to extensive cocoa production, logging, bushfires, and small-scale mining, Juabuso HIA experiences one of the highest deforestation rates in Ghana (Forestry Commission 2017; Nasser et al. 2020). Trend analysis has shown that annual forest losses within the Juabuso-Bia landscape almost doubled from 2941 ha/yr in 2014 to 4575 ha/yr in 2018 (Kessler et al. 2020).

### Data Collection Methods and Sampling

We applied and extended the Q-methodology to include semi-structured interviews and focus group discussions to examine stakeholder perceptions of the highly prioritized NCBs of Ghana’s Cocoa REDD+. In addition to survey and focus group data, we also gathered information on the activities of the HIA by attending the Bia sub-HIA’s annual review meeting and Manzam’s CREMA constitution drafting session.

Data for the study was collected between November 2021 and January 2022, with a total of 108 respondents participating in the research. We used purposive sampling to select the Juabuso-Bia HIA and study participants (Duan and Hoagwood 2015; Zabala et al. 2018). The non-probability (non-random) sampling technique was used to target HIA/CREMA communities and active participants in Ghana Cocoa REDD+ who had the requisite knowledge about the subject. The largest group of respondents consisted of cocoa farmers, followed by HIA/CREMA executives, government officials, non-governmental organizations (NGOs), academic researchers, cocoa purchasing clerks, and farm laborers (Table 4). Sixty people from all stakeholder categories were interviewed, and half of them also participated in the Q-exercise. Eighteen respondents participated in three focus group discussions. Respondents came from 12 communities in six CREMAs within and outside the Juabuso-Bia HIA. Table 4 gives an overview of the sample for Q-methodology, the semi-structured interviews, and the focus groups, split between the types of respondents.

**Table 4:** Category and Numbers of Respondents by Data Collection Method

Category of respondents	# Q-sort respondents	# Interview respondents	# Focus group respondents
Cocoa farmers	10	17	10
State officials (Forestry Commission and COCOBOD)	4	5	3
HIA/CREMA executives (male)	4	10	0
HIA/CREMA executives (female)	4	7	5
Non-governmental organizations	3	7	0
Academic researchers	2	4	0
Licensed Cocoa Buying Companies (LBCs)	0	4	0
Cocoa purchasing clerks (PCs)	2	4	0
Farm laborers	1	2	0
Total	30	60	18

Below, we provide more details on the data collection methods, which were pre-tested and refined in a pilot study conducted in the Debiso CREMA in November 2021.

#### Q-methodology

Q-methodology, developed in the 1930s by William Stephenson (Brown 1980), was used to analyze stakeholder perceptions of the capacity of Juabuso-Bia HIA to generate governance, economic and social NCBs. Q-methodology combines qualitative data collection (ranking of statements) with quantitative research techniques in a structured manner to identify different perspectives or common viewpoints among stakeholders (Zabala and Pascual 2016; Zabala et al. 2018). Originally applied in psychology, Q-methodology found broader applications, extending into political science and various other disciplines (Watts and Stenner 2012). In forest governance and conservation, the methodology has been used before to assess perceptions of the effectiveness of Forest Law Governance and Trade in Ghana (Adams et al. 2021), community forestry in Cambodia (Nhem and Lee 2020), and the role of multistakeholder platforms in Zambia (Siangulube 2024, this issue). The underlying assumption of Q-methodology is that viewpoints are subjective and can be shared, measured, and compared (Fontein-Kuipers 2016). By employing Q-methodology, we sought to bridge the gap between quantitative and qualitative research and gain insights into policy from a social perspective (Sumberg et al. 2017; Zabala et al. 2018).

The first step in Q-methodology is developing a “concourse” encompassing the “full opinion spectrum” from which the Q-set (the representative set of statements) (Churruca et al. 2021) on HIA and NCB are derived. The concourse of this study was drawn from peer-reviewed publications, technical reports, and ethnographic studies on REDD+, HIA, NCBs, and the cocoa value chain. A review of these technical reports and REDD+ studies produced 81 statements. We categorized the 81 statements according to subtopics (governance, economic, and social NCBs) and chose a balanced number from all subtopics. To ensure the validity and relevance of the Q-set, three experts from Ghana’s academic and forest sectors reviewed the statements. This process improved the quality and comprehensiveness of the statements, resulting in a final list of 58 statements (see Appendix 1).^4^

Next, we selected the participants (forming the P-set) for the Q-sorting exercise. For this study, we selected participants representing diverse groups and viewpoints using a purposive snowball sampling approach. Participants included a range of stakeholders, including 30 individuals selected from within the Juabuso-Bia HIA and from organizations working with the Ghana Cocoa REDD+ or engaged in studies on the cocoa landscape (Table 4). The selected participants have knowledge of the Ghana Cocoa REDD+ or actively participate in the program activities. The size of the P-set accommodated the broad perspectives of the diverse stakeholder population for the study. The P-set is therefore considered sufficient for capturing different perspectives, as Q-methodology focuses on understanding the subjective expressions of participants rather than achieving generalizability (Simpson and Hill 2020).

The selected participants were given the Q-set of 58 statements in the form of indexed cards and were asked to sort and rank the statements on a nine-columned grid (−4 to +4) with a quasi-normal distribution based on their subjective views on the HIA and NCBs. The range interval of 9 provided ample space for participants to express their opinions and ranged from “I strongly disagree” (−4) to “I strongly agree (+4)” (Nhem and Lee 2020). Participants completed the Q-sort in response to the provided instructions, prioritizing their views and perceptions about governance, social, and economic NCBs under Ghana Cocoa REDD+. To accommodate cocoa farmers, the statements were verbally translated into the local language (Twi) prior to sorting. Post-sort interviews were conducted with participants of the Q-exercise. Due to COVID-19 restrictions, five Q-sorts (the outcome of the sorting exercise) were sent via email. Photos of all the Q-sorts were taken, and the data was extracted into an Excel database.

One criticism of Q-methodology is the limitation of Q-statements in fully describing the range of perspectives on the subject under study (van Exel and de Graaf 2005). To address this limitation, we followed the approach proposed by (Kirschbaum et al. 2019) to include post-Q-sort interviews as additional probes. We also mitigated the potential bias in participant selection by employing snowball sampling and seed diversity techniques. These techniques introduced another level of diversity in the demographics of participants (Zabala et al. 2018) and ensured that the initial set of respondents was sufficiently varied for the Q-exercise (Kirchherr and Charles 2018).

#### Extending the Q-methodology

This study combines Q-methodology with semi-structured interviews and focus group discussions for deeper and contextualized insights into perceptions on how the HIA can potentially facilitate the generation of governance, social, and economic NCBs to stakeholders within the cocoa landscape. This combination also helps to validate the perspectives from the Q-sorts. The focus of Q-sort interviews on participants’ logic for sorting, perceptions, and attitudes to the phenomenon under study (Gallagher and Porock 2010; Robin 2005) may limit their responses in relation to the wider context of Ghana Cocoa REDD+, external factors, and the general conditions in the HIA. Participants’ responses in Q-sort interviews may not be reflective of what they had originally intended because they are influenced by the statements sorted during the Q-exercise.

The semi-structured interviews (N = 60, see Table 4) addressed the perceived reliability issues of Q-sorts identified by van Exel and de Graaf (2005) and helped deepen the knowledge generated by the Q-sort. They focused on qualitative individual perceptions of HIA establishment and governance, inclusion and participation, particularly of women and vulnerable groups, and land and tree tenure. The interviews also gathered quantitative livelihood information. Half of the sampled respondents had also participated in the Q-exercise; the other half of the sample did not because the P-set of 30 participants was sufficient to generate a range of perspectives on the Ghana Cocoa REDD+ NCBs in the Juabuso-Bia HIA landscape.

#### Focus Group Discussions

We conducted three focus groups to observe arguments and leverage group dynamics and deliberations to gain a further in-depth understanding of perceptions among stakeholders (Nyumba et al. 2017). One focus group discussion was held with five female farmers and CREMA executives; a second focus group with ten male farmers and CREMA executives, both from the HIA; and the third focus group with three senior staff members of COCOBOD in Accra.

Topics discussed in the focus groups included landscape governance, cocoa production, livelihoods, and tenure, which are among the highly prioritized NCBs under Ghana Cocoa REDD+. Specific topics discussed in the COCOBOD focus group were cocoa production, improvements in farmers’ livelihoods under the program, and concerns raised by farmers during interviews we had with them. The focus groups with female farmers covered challenges in cocoa production, tenure security, and participation in governance activities in the landscape, while the issues of low cocoa yield, availability of farm inputs, and low incomes were the major topics discussed in the focus groups with male farmers.

### Data Analysis

#### Q-methodology Data Analysis

We used the qmethod package in R with a graphical interface developed by Aiora Zabala (Version 1.8.1) to convert the Q-sorts into factors. This process was broadly guided by the work of Zabala (2014), Zabala and Pascual (2016), and Zabala et al. (2018). We constructed a matrix in an Excel database where rows represent statements, columns represent the respondents, and the cells represent the scores each respondent gave each statement during the sorting process. The entire set of scores per respondent is referred to as the Q-sort. This data underwent cleaning to correct errors. We also verified that the sum of the values assigned to a statement by each participant was zero to ensure data integrity. Subsequently, the Excel database was transformed into a .csv file and imported into the R programming environment for further analysis.

Using principal component analysis (PCA), a multivariate data reduction technique, all the Q-sorts completed were compared and grouped into a few representative responses. As in standard PCA, four factors were chosen from the components based on specific criteria of an eigenvalue greater than 1 and the percentage of explained variance. Following Zabala et al.’s (2018) recommendation, a varimax rotation was applied to these selected factors. This process aimed to obtain clearer and more interpretable results in a table displaying factor loadings attributed to participants. Notably, participants’ loadings in the factors were flagged with a 95% confidence level.

Subsequently, a series of tables were generated, featuring Z-score rankings for each statement, and identifying distinguishing statements within each factor. These tables served as the foundation for constructing a set of reconfigured Q-sorts, one for each factor, based on the composite and weighted Z-scores from participants defining a particular factor. In this study, the interpretation of factors is based on a combination of the statement scores, statements that are both distinctive and with the highest or lowest scores or statements at the extremes, qualitative data collected from the respondents during the Q-sort, and the researcher’s experience and understanding of the elements being studied (Zabala 2014; Zabala et al. 2018). We also selected statements with the highest and lowest scores, following the procedure applied in studies by Nhem and Lee (2020) and Simpson and Hill (2020) for interpretation. Each of the four identified factors was given a meaningful label and described subsequently.

#### Interview and Focus Group Data Analysis

Interviews and focus groups in English were transcribed using Otter.ai 2.0, while those in Twi were transcribed manually. Building on the work of Kiger and Varpio (2020) and Braun and Clarke (2006), we employed a thematic analysis approach to identify patterns and analyze data from the transcribed information. Within the transcribed data, we identified and coded items such as “landscape governance”, “vulnerable groups and participation”, “governance instrument”, “land and tree tenure”, and “cocoa production and poverty”. We then grouped related items from the coding exercise (based on patterns, repetitions, or similarities) to generate the following themes: “the potential and limitations of enhanced forest governance through the HIA”, “the limits to poverty alleviation”, “limits to inclusion and participation”, and “tenure rights limitations”. We ensured that the themes selected aligned with the transcribed data and research questions of this study. We further constructed a narrative of each of the four themes, based on the interview and focus group results, to provide deeper insights into the perspectives generated from the Q-exercise.

## Results

This section first presents the results of the Q-methodology for a general overview and perspectives of stakeholder perceptions. It then deepens the insights based on the interviews and focus group discussions. Characterization of Perspectives of the HIA and Ghana Cocoa REDD+ using Q-methodology

The rotation revealed four factors, representing distinct viewpoints of actors on Ghana Cocoa REDD+ and NCBs (see Appendices 2 and 3). The factors represent 38% of the variability of the Q-sort (see Appendix 4), which is within the acceptable range of 35–40% used by Nhem and Lee (2020). A positive factor loading indicates shared subjectivity, while a negative loading indicates rejection (see Appendix 2). We also analyzed and incorporated distinguishing and consensus statements in the description of the perspectives (see Appendix 9). We present the characterization of the perspectives below.

### Perspective 1: Ghana Cocoa REDD+ Strengthens Sub-National Forest Governance

Based on distinguishing statements, perspective 1 explains 13% of the study variance, and 10 participants loaded significantly on this perspective (see Appendix 4). Participants championing this perspective are mainly community members (HIA/CREMA executives, farmers, and purchasing clerks), but also representatives of a government agency and an NGO agreed to this perspective. The highest loading of 0.72 was associated with a female HIA/CREMA executive (see Appendix 2). This perspective acknowledges the diminishing size and declining value of forests in the landscape (23; 3; 1.13)^5^ and the detrimental effect of climate change on cocoa production (4; 4; 1.48). The Ghana Cocoa REDD+ in the HIA is perceived to equip communities with the necessary tools to cope or adapt to the impacts of climate change (2; 4; 2.19) and could recognize and involve communities (CREMAs) in decision-making processes and program implementation (10; 3; 1.39). Ghana Cocoa REDD+ could also foster effective collaboration among state agencies, the private sector, and civil society (44; 3; 1.32) and has adequate measures to address natural resource conflicts (37; 4; 1.65). Notably, the program poses no threat to livelihoods and employment opportunities in the CREMAs (35; −3; −1.32). The program may not adversely affect the developmental needs of the HIA and CREMAs (50; −3; −1.42) and is not perceived to have the potential to bring conflicts among stakeholders (46; −4; −1.92). However, the program’s benefit-sharing scheme does not adequately address the exclusion of women and vulnerable groups (47; −3; −1.29). Appendix 5 provides the ranking values for perspective 1.

### Perspective 2: Prospects for Poverty Alleviation but Not for All

Perspective 2 explains 9% of the total variance for the study, and seven participants loaded significantly (see Appendix 2). This perspective is dominated by farmers. A farmer and a farm laborer loaded highest at 0.66 each and a researcher at −0.57 (see Appendix 4). This perspective acknowledges that the Ghana Cocoa REDD+ is important for alleviating poverty among cocoa farmers (28; 3; 1.18) and that additional livelihood interventions initiated by the Ghana REDD+ are adequate for achieving a decent standard of living (30; 4; 1.83). However, the program also poses potential threats to livelihoods and employment opportunities (35; 3; 1.07), and there are concerns that the expected benefits that farmers would receive from the program are less than what they currently earn from their existing land use practices (41; −3; −1.53). Participants perceive that Ghana Cocoa REDD+ excludes women and vulnerable groups from benefit sharing (47; −3; −1.89), and land and tree tenure regimes also marginalize these groups and limit their access to performance-based payments (54; −3; −1.51). The program has limited opportunities for women and migrants with no land-owning rights to benefit from carbon credits (55; −3; −1.37) but has adequate safeguards ensuring that CREMA communities are not worse off through the program (9; 3; 1.26). There are increased activities of beneficial insects (pollinators) in support of cocoa production (20; 4; 1.42), but there is a perceived underemphasis on the promotion of organic cocoa production as compared to conventional cocoa within the program (1; 3; 1.03). Participants hold the view that the role of intermediaries in the sale of cocoa beans does not necessarily reduce farmers’ incomes (29; −4; −2.24), and commodity companies have done much to address cocoa-driven deforestation (25; −4; −1.96). See Appendix 6 for the ranking values.

### Perspective 3: Effective Policy Design but Poor Stakeholder Collaboration and Selective Inclusion and Participation

Perspective 3 explains 8% of the total variance for the study, and four participants loaded significantly (see Appendix 4). Community members and an NGO representative dominated this perspective. Two farmers from Juabuso-Bia loaded highest at 0.82 and 0.76, and an NGO representative at −0.37 (see Appendix 2). The discourse under this perspective suggests that current climate change policy effectively addresses REDD+ implementation and practice (40; 3; 1.28). Under the Ghana Cocoa REDD+, seedlings for climate-smart agriculture are readily provided in a timely manner (3; 4; 1.97), but there have not been increased activities of beneficial insects (pollinators) to support cocoa production (20; −4; −2.06). There is clarity regarding the institutions responsible for each component of the Ghana Cocoa REDD+ (51; 3; 1.43), and this group of participants agrees that enhanced livelihoods will lead to emission reductions (34; 4; 2.12). The Ghana Cocoa REDD+ does not recognize community participation in decision-making processes to address deforestation in the HIA (10; −3; −1.62). The program represents an important intervention in poverty reduction among cocoa farmers (28: 4; 1.67), but opportunities for women and landless migrants to benefit from the program are limited (55; −3; −1.22). Over the past five years, there has been an observable increase in cocoa farm sizes with the intention of increasing cocoa yields (52; 3; 1.31), and participants do not agree that the cost of tree rights registration would favor wealthy cocoa farmers in terms of carbon payments (57; −4; −2.25). This group of participants strongly agrees that there is inadequate access to fresh water for domestic purposes (24; −4; −1.65) and acknowledges that climate change negatively influences cocoa production (4; 3; 1.26). See Appendix 7 for the ranking values.

### Perspective 4: Land and Tree Tenure Rights and Resource Access are Not for the Poor

Perspective 4 relates to land and tree tenure rights and natural resource access, and 4 participants loaded significantly. This perspective explains 5% of the total variance of the Q-sort and is dominated by government officials (see Appendix 4). Two government officials loaded significantly at 0.71 and 0.54, and one farmer loaded significantly at 0.57 (see Appendix 2). This perspective proposes that the inadequacy of well-defined land and tree tenure rights is linked to tree losses (56; 3; 1.35), and there is also evidence of increased timber harvesting within the HIA/CREMA (22; 4; 2.04). Furthermore, the abundance of non-timber forest products has declined (17; −3; −1.19), and climate change is influencing cocoa production negatively (4; 4; 2.44). Benefits from Ghana Cocoa REDD+ are not perceived to be higher than those from farmers’ current land-use practices (41; −4; −1.6), and participants also prefer cash benefits from result-based payments (45; 4; 1.59). Participants feel apprehensive when accessing ecosystem services within the HIA/CREMA (26; −3; −1.23) and agree to the requirement of permits to collect non-timber forest products from the HIA/CREMA (12; 3; 1.2). Engagement with communities is perceived to have been inadequate (11: −4; −1.76), and participants acknowledge that the program has not resulted in a sustainable increase in cocoa production over the past five years (5; −3; −1.23). Curbing deforestation through cocoa has not compromised the development needs of the HIA/CREMA (50; −4; −1.66), and women farmers’ incomes have increased (31; 3; 1.09) under the program. See Appendix 8 for the ranking values.

### Consensus Statement

We identified one consensus (overlapping) statement where participants ranked similarly across all perspectives. Participants agree that the CREMA governance model offers a platform for stakeholders to dialogue and collaborate on REDD+ implementation (43) (see Appendix 9). Other areas of consensus between at least two perspectives include statements 28 (perspectives 2 and 3) and 41 (perspectives 2 and 4) (see Appendix 1 for the statements).

### Enriching the Findings: Insights from Interviews and Focus Groups

The Q-analysis revealed four major themes related to governance, social and economic NCBs from Ghana Cocoa REDD+, of which three relate to governance NCBs: the potential and limitations of enhanced forest governance through the HIA; inclusion and participation; and land and tree tenure rights. One theme relates to social and economic NCBs: the limits to poverty alleviation. The subsections below provide more in-depth insights and enrich the findings on these themes based on the semi-structured interviews and focus groups.

#### The Potential and Limitations of Enhanced Forest Governance through the HIA

The interviews and focus groups with farmers and consortium partners reveal that the Juabuso-Bia HIA has initiated meaningful discussions on cocoa-induced deforestation and offered options for a collective response. The HIA has also incentivized the private sector to invest in the HIA, including the CREMAs, by supporting the development of by-laws, implementing alternative livelihood interventions, strengthening the HIA governance structures, and training HIA executives in landscape management. Through interviews, HIA executives suggested that the HIA governance framework has facilitated collaboration with cocoa companies and NGOs to improve forest governance in the landscape. A sub-HIA executive indicated that “Juabuso-Bia HIA has facilitated collaboration with other sub-HIAs in reducing unsustainable land use practices.” However, NGOs indicated in interviews that the HIA is not taking the lead in facilitating collaboration among sub-HIAs; rather, this is being left to the Forestry Commission, NGOs, and cocoa companies.

We deduced from the interviews and focus group discussions with respondents from the Forestry Commission and NGOs that in establishing the HIA to strengthen landscape governance, several quasi-legal governance instruments were developed. For example, both CREMAs and the sub-HIAs have constitutions, management plans, and by-laws. From the interviews with the NGOs, we gathered that some of the governance instruments for the CREMAs that existed prior to the HIA have not been revised to reflect the HIA’s new structure and functions. Despite the development of quasi-governance frameworks, we found through interviews that CREMA executives and field-level officers of the consortium partners were not familiar with the HIA structures and most of the governance instruments. It also emerged from the focus group discussions that poor dissemination of HIA activities has led to low awareness of the HIA and its activities in the cocoa landscape. Due to the low local awareness of the HIA, a representative of the Forestry Commission posited that “very few people involve themselves in HIA activities.”

Interviews with the Forestry Commission indicate that the limited inclusion of communities in the HIA is partly due to financial constraints and partly due to the unwillingness of some communities to be part of the HIA governance system. The Forestry Commission was of the view that they had provided all necessary information to communities by applying principles of Free Prior and Informed Consent (FPIC) in the REDD+ program. However, interviews with NGOs revealed weaknesses in community entry processes and a lack of efforts by the Forestry Commission to sustain communities’ interest in the Ghana Cocoa REDD+ program. An example mentioned by NGOs includes inadequate consultation with key stakeholders in some of the communities, despite the claim of the Forestry Commission to have fully applied FPIC principles in the REDD+ program. This appears to be affecting the geographical connectivity and capability of the HIA to build a network of communities to support sustainable governance practices. In the interviews with the HIA Management Board and sub-HIA executives, financial constraints stand out as a major challenge. A Board member stated, “We are unable to generate funds to support our activities except to depend on NGOs and cocoa companies operating in the landscape.”

The interviews with representatives of NGOs, the Cocoa Board, and the Forestry Commission involved in Ghana Cocoa REDD+ suggest that the functional challenges faced by the HIA resulted from the rapid establishment of governance structures. Issues such as dysfunctional leadership, elite capture, poor finances, and undemocratic tenets of the existing CREMAs were not addressed before establishing the HIA. A partner in the consortium recounted, “The pressure was so high from the Ghana National REDD+ Secretariat and the World Bank to complete the whole governance structure… and the truth is, yeah, we did it, but I would not call it best practice”.

Law enforcement and compliance in the landscape are perceived to be ineffective. Through interviews with farmers and personal observations, we find that very little is being done to relocate owners of “illegal farms” in the landscape. Illegal logging and mining activities are also rife in the Krokosua Forest Reserve and other areas within the cocoa landscape. According to the sub-HIA and CREMA executives, monitoring teams in the HIA lack basic working equipment such as personal protective equipment and transport for field activities. The CREMA executives also suggested that the situation appears to be further compounded by political interference and the complicity of some traditional leaders who are involved in illegal forest activities.

In analyzing the interviews with the Forestry Commission and the cocoa company Touton on the contribution of cocoa companies to forest governance, we found that the Juabuso-Bia HIA and the first set of activities under Ghana Cocoa REDD+ were funded by Touton under the Partnership for Productivity, Protection, and Resilience in Cocoa Landscapes project (3PRCL). The 3PRCL was a three-year project targeting the production of deforestation-free cocoa beans. This was probably the first formalized relationship between a cocoa company and the HIA/CREMAs. Subsequently, other companies operating in the landscape appear to have contributed to improving HIA governance. Participants in focus groups and interviewed farmers and NGOs perceive, however, that cocoa companies tend to exaggerate their contributions to the REDD+ program, misrepresent or conceal information about their cocoa bean sourcing, and engage in double reporting. NGOs suggest that the double reporting is likely a result of the cocoa companies’ involvement in both the Cocoa Forest Initiative and the Ghana Cocoa REDD+.

#### The Limits to Poverty Alleviation

A focus group discussion with COCOBOD confirmed that the main economic NCB from Ghana’s Cocoa REDD+ program is the increase in cocoa production on existing farms. This is expected to be achieved by promoting cocoa intensification and climate-smart cocoa practices. The adoption of these practices by farmers to increase farm productivity would reduce the incentives for farmers to clear forest land for cocoa cultivation. The participants from COCOBOD also assumed that increased cocoa production would lead to increased incomes and a possible reduction in poverty among farmers in the Juabuso-Bia landscape. Interviews with the consortium partners confirm they have established demonstration farms, provided peer-learning experiences to farmers, and supported cocoa agroforestry and farm rehabilitation activities, recognizing their responsibilities in leveraging the HIA to support the livelihood activities of cocoa farmers.

A focus group discussion with COCOBOD and interviews with academic researchers and farm laborers suggested that capacity building for farmers in integrated pest management, crop and soil/water management, and agrochemical usage has been taking place. Interviews with farmers also confirmed that they had received farm input supplies, economic and shade trees, and training in climate-smart cocoa through the HIA structures and farmers’ cooperatives. Farmers expressed the expectation that this would help increase yields on their existing farms. In a focus group discussion, COCOBOD expressed its commitment to supporting farmers with adequate farm inputs to increase cocoa yields but acknowledged that they are limited financially to provide sufficient levels of support and training.

Cocoa farmers and COCOBOD expressed in focus group discussions and interviews that the uptake of climate-smart cocoa has been seriously hampered by inadequate amounts of farm input supplies, conflicting communication on climate-smart cocoa, poor cocoa extension services, unfavorable financing schemes and the abandonment of uncompleted cocoa rehabilitation farms. In relation to the latter, a CREMA executive recounted, “A farmer cleared all his 8-acre cocoa farm in anticipation of having his farm rehabilitated but never received the needed support. We now call the farm ‘airplane park’.

In relation to the amount of farm input supplies, interviews and focus group discussions with farmers, farm laborers, and COCOBOD all confirmed that farmers are supplied with inadequate quantities of Cocoa Board-approved fertilizers and pesticides. The farmers interviewed also expressed serious concerns about receiving supplies irregularly from COCOBOD and the companies. They further expressed their dissatisfaction with the low productivity of their farms and its effects on their incomes: A female farmer explained, “Years ago, when we harvested cocoa from our farms and heaped the beans, and a person sitting by the heap raised their hand, a person on the other side of the heap could not see the raised hand. That is not the case anymore.”

Other concerns of farmers about the ability of Ghana REDD+ to alleviate poverty, expressed through interviews, include the low seasonal producer price, tenure insecurity, and the short-changing of cocoa farmers by cocoa purchasing clerks. Cocoa purchasing clerks, in turn, expressed dissatisfaction with the delay in the release of funds by cocoa companies for cocoa purchases. A clerk stated, “We are not able to pay farmers promptly when there is a delay in the release of funds for cocoa purchases.” Based on these concerns and challenges, a cocoa farmer claims that “Currently, cocoa farming is unattractive, and if you want to identify some of the poorest people in Ghana, then it is cocoa farmers.” In addressing these concerns, cocoa companies explained that they introduced some income diversification enterprises (mushroom farming, beekeeping, fish farming, grasscutter rearing, and soap making) to supplement farmers’ income.

Interviewed cocoa companies revealed that they prioritize support service delivery models and structures outside the HIA to provide a variety of services (such as farm inputs) to their registered farmers rather than to registered farmers involved in the Ghana Cocoa REDD+. Most interviewed consortium partners expressed frustration about the quality of collaboration among the consortium, leading to reduced cocoa production on existing farms.

Despite efforts through Ghana Cocoa REDD+ to reduce incentives that drive the expansion of cocoa farms into forest areas, interviews with farmers indicate that in the past five years, they have increased their farm sizes by an average of 3-4 acres. These increases in farm size were undertaken purposely to increase cocoa production and involved the clearing of both reserve and off-reserve forest areas. According to most farmers in both interviews and focus group discussions, unprecedented cocoa production levels recorded in the 2020/21 season were mostly due to new farms and the smuggling of cocoa into Ghana along the Ghana-Côte d’Ivoire border.

#### Limits to Inclusion and Participation

Based on the interviews and focus group discussions with actors, the inclusion and participation of community actors in HIA activities can be described as tokenism. Some farmers indicated that they are not fully represented at the consortium meetings where key decisions on HIA governance are made. In the focus group discussions, farmers expressed concerns that their interests are rarely considered in the Ghana Cocoa REDD+ program except when it serves the interest of the consortium partners. Several CREMA executives also expressed frustration at what they see as the inadequate flow of information from the HIA Management Board to the CREMAs.

Interviewed farmers and NGOs questioned the role of the Feedback and Grievances Reporting System (FGRS) in providing a platform for participation by addressing the concerns of stakeholders in the Ghana Cocoa REDD+ program. The FGRS is a REDD+ safeguard mechanism for addressing grievances and potential conflicts in Ghana Cocoa REDD+ and is administered by the Forestry Commission. Farmers and NGO representatives questioned the low visibility of the FGRS committee in the landscape and raised concerns about its composition as a neutral arbiter in addressing farmers’ grievances.

Our interviews with the HIA Management Board revealed that women make up 27% of the Board’s membership, while more women have been appointed to lower levels of the HIA governance system, where they have less influence on decision-making. According to the Board, they have not achieved the objective of 50% female representation. The separate focus group discussions among male and female farmers suggested that the continued low participation of women in HIA/CREMA activities is still a challenge due to gender norms.

Nonetheless, interviews with NGOs revealed that the HIA has contributed to building women’s capacity to conduct farm surveys relating to cocoa farming activities and collect biodiversity data. Also, interviews and focus group discussions confirmed that, due to the various sensitization activities in the cocoa landscape by the consortium partners, more women are nurturing the desire to put themselves up for leadership positions. A case in point is the bold statement made by a female CREMA executive during an interview that “I can become a CREMA chairperson”.

#### Tenure Rights Limitations

Farmers, farm laborers, and the members of the consortium partners confirm in interviews that the uptake of economic/shade tree planting on farms in the cocoa landscape is increasing. Farmers explain their rationale for planting economic and shade trees in interviews as “Trees call rains”; “Where we planted *oframu*, we ‘ve had more cocoa pods”; and “Shade trees attract birds into our farms to control insects.” Interviews and focus group discussions with CREMA executives revealed that some CREMAs have established community plantations with communal tenure rights, with funding for the initial labor costs provided by NGOs. Farmers involved in the modified taungya system have also replanted depleted forests, where benefits would be shared among farmers, the Forestry Commission, landowners, and local communities.

From the interviews with farmers and academic researchers who work in the cocoa landscape, we understand that the incorporation of shade and economic trees on farms is currently hampered by the erratic supply of seedlings from the Forestry Commission, NGOs, and cocoa companies. Another challenge is the piecemeal approach to tree rights registration, which has led to a scaling down of tree planting efforts. Due to the challenges with tree rights registration, “Some farmers in Yawmatwa are cutting down their planted trees to prevent timber companies from harvesting them”, according to an NGO representative in the Juabuso-Bia HIA.

#### Complementary Findings and Contradictions in the Outcomes of Our Methods

All three methods (Q-methodology, semi-structured interviews, and focus groups) generated outcomes to suggest that the forests in the Juabuso-Bia HIA landscape were diminishing in size and value but that the Ghana Cocoa REDD+ has the potential to reverse this trend – supporting the findings on the potential of enhanced sub-national governance through the HIA. Community participation and collective decision-making are particularly supported by the constitution of all the sub-HIAs, which focus on the importance of bringing together chiefs, farmers, and other stakeholders within the landscape to collaboratively address deforestation.

In relation to inclusion and participation, the sub-HIA constitution also establishes the quota system, allowing for the representation of women and minority groups across the HIA hierarchy. However, women are not yet sufficiently represented at the different management levels of the HIA, although more women are engaged at the CREMA level than at the level of the Sub-HIA Executive Committees and the HIA Management Board.

Although there was consensus among the methods that the CREMA provides a platform for engagement in natural resource management, the interviews and focus groups added a deeper understanding of some of the limitations associated with HIA governance. Issues of rapid implementation of the HIA structure and the carry-over of prior problems of elite capture, dysfunctional leadership, and inadequate democratic practices of the existing CREMAs are perceived to limit the effectiveness of the HIA to facilitate the implementation of Ghana Cocoa REDD+. Also, the move toward a top-down approach and a recentralization of decision-making has affected the engagement of CREMA communities.

Interviews provide a general picture that collaboration between the HIA and the consortium partners still needs improvement to facilitate effective landscape governance. Outcomes from the three methods also indicate that the Ghana Cocoa REDD+ program is likely to struggle to improve farmers’ livelihoods and address poverty because of limited efforts to strengthen land and tree security for cocoa farmers and not providing a sufficient and stable supply of farm inputs, effective extension services, and cocoa rehabilitation to farmers.

While the Q-sort largely suggests that the HIA and CREMA governance framework fosters community participation, the interviews and focus groups give ample details of the difficulties communities have in effectively participating in the HIA, the lack of dissemination and awareness of HIA activities among communities, and the failure to address issues of dysfunctional leadership, elite capture, and undemocratic tenets of existing CREMAs prior to the creation of the HIA. The interviews and focus group discussions indicate that community participation is further weakened with the expansion of the CREMAs to HIA status.

## Discussion

This study sought to examine stakeholder perceptions of the HIA to facilitate the generation of governance and economic and social NCBs. This section positions the main findings related to the four perspectives on Ghana Cocoa REDD+ identified through Q-methodology in the broader literature, reflects on the implications, and discusses the study’s methodological contribution and research limitations.

### The Role of Ghana Cocoa REDD+ in Strengthening Sub-National Forest Governance

The Juabuso-Bia HIA, evolving from a community-based natural resource management framework (CREMA) to broader partnerships, exhibits several environmental governance domains (Lemos and Agrawal 2006): globalization, decentralization, market and individual incentives-based, and cross-scale governance. This complexity necessitates hybrid governance modes such as co-management and multisector partnerships to address environmental degradation effectively. However, the recentralization of landscape governance through the HIA faces policy incoherence. For example, the HIA is not mentioned in important national policies such as the Wildlife Resources Management Bill (2022), the Ghana Forestry Development Master Plan (2016–2035), and the Ghana REDD+ Strategy (2016–2035). This policy incoherence could result in constrained resource allocation to the HIA, hindering effective landscape governance. We postulate that the inherent limitations in capabilities, capacities, and resource allocation for natural resource and landscape governance, which rendered CREMAs ineffective, remained unaddressed.

Moreover, multi-actor governance may result in symbolic violence, i.e., powerful actors benefiting from unchallenged power and access to resources (Mustalahti et al. (2020), as sub-national governance initiatives do not always imply meaningful powers. Power imbalances between the consortium partners and the HIA executives limit the independence of the HIA in decision-making processes, potentially leading to power struggles rather than power-sharing (Makatta et al. 2015) and accelerated natural resource degradation rather than improved conservation. Despite the importance of multi-stakeholder involvement for favorable REDD+ outcomes in an otherwise fragmented governance arena (Gupta et al. 2016), the ability of the HIA to connect with actors at multiple levels is restricted due to limited autonomy in decision-making processes, financial constraints, restricted geographical area, and weak law enforcement.

Notwithstanding its intent, the Juabuso-Bia HIA faces a “capability trap” (Pritchett et al. 2013) that hinders its capacity to generate governance and economic NCBs. Weak governance structures due to hasty implementation, poor application of Free, Prior, and Informed Consent, and poor coordination among actors undermine landscape governance, as also illustrated in other studies (Korhonen-Kurki et al. 2014; Saeed et al. 2017).

### Prospects for Poverty Alleviation

The second perspective critiques the effectiveness of measures to alleviate poverty through climate-smart cocoa production, a concern echoed by farmers in the Q-sorts analysis. Despite commitments from institutions like COCOBOD to support farmers with inputs, extension, and training, financial constraints, abandonment of initiatives on the ground, and poor communication limit their ability to double productivity to 800 kg/ha/yr (Forestry Commission 2020), risking Ghana Cocoa REDD+ to achieve economic and social NCBs. Recurrent losses in COCOBOD’s operations (COCOBOD 2018, 2019), compounded by unrelated expenditures such as for funerals, further hinder support for farmers.

The perceived inadequacy of current measures, alongside low yields and pricing mechanisms, threaten farmer commitment to forest conservation efforts under the Ghana Cocoa REDD+ program. The Living Income Differential (LID), implemented by the government of Ghana in 2019, adding 400 USD per ton to all cocoa sales to raise cocoa farmers’ income (Adams and Carodenuto 2023), the EU Corporate Sustainability Reporting Directive (European Union 2022), and the UK Environment Act (Parliament of the UK 2021) are positive steps toward improving farmers’ livelihoods and social and environmental conditions and support the intention of Ghana Cocoa REDD+ to generate economic and social NCBs. However, there is a need for transparency and accountability to ensure that a significant amount of the LID is passed on to farmers and cocoa farming communities (Fountain 2022).

### Inclusion and Participation

The third perspective emphasizes the importance of inclusive policies and collaboration in landscape governance. Despite intentions for inclusive participation within the CREMA and Ghana Cocoa REDD+ program (Murray et al. (2019) through sub-HIA constitutions and the framework agreement of implementation and potential socioeconomic NCBs, implementation falls short, excluding actors like miners and women due to limited recognition and parity in the agenda-setting of Ghana Cocoa REDD+ (McDermott et al. 2022). Efforts to mainstream gender equality in HIA governance fall short of bridging the equity gap and can be qualified as tokenism (Arnstein 1969). Women’s limited participation in HIA governance is attributable to gender norms, power imbalances, and women’s poor negotiation skills. Similarly, migrant farmers are underrepresented in HIA and Ghana Cocoa REDD+ governance. Genuine discussions and actions characterized by FPIC are recommended to increase the representation of marginalized actors in the HIA administrative structure to prevent REDD+ initiatives from reinforcing existing disparities (Larson et al. 2018; Schmitt and Mukungu 2019). While the consortium partners have made investments in improving landscape governance, their fragmented approach hampers effective coordination, hindering the generation of governance and economic NCBs. Governments rather than the private sector should lead REDD+ initiatives to prevent conflicting interests and ensure oversight. Participatory decision-making is crucial to sustaining emission reductions when performance-based payments are suspended, as happened in Brazil (Carrilho and Wunder 2023) and Guyana (Roopsind et al. 2019). Payment for Ecosystem Services (PES) programs like REDD+ based on inclusive and participatory decision-making processes foster beneficiaries’ intrinsic motivations to manage ecosystems sustainably. Improved inclusive participation can enhance actors’ motivation to sustain deforestation and emission reductions beyond 2034 when the Ghana Cocoa REDD+ result-based payments come to an end.

### Tenure Rights

The fourth perspective highlights disparities in land and tree tenure and resource access, particularly women and vulnerable groups who may not benefit from Ghana Cocoa REDD+’s prioritized NCBs. Efforts to enhance tenure security for women and influence land-use practices in the HIA are lacking. Similar challenges have been observed in Peru, where REDD+ struggled to influence land use and landscape governance due to more lucrative economic alternatives like mining, mirroring risk in the HIA with illegal mining (Rodriguez-Ward et al. 2018).

Increased planting of economic and shade trees on farms cannot be wholly attributed to Ghana Cocoa REDD+, with past initiatives like the Forest Investment Program having contributed to the progress made (Selvakumar and Suksangium 2023). However, farmers still face limited control (O’Sullivan et al. 2018) and tenure equity issues (Nasser et al. 2020) over trees planted on their farms because the Tree Tenure and Benefit Sharing Framework (MLNR 2016) has not adequately addressed tree tenure challenges. The piecemeal approach to tree rights registration could hinder the scaling-up of tree planting efforts as “complex, multiple and unclear tenurial arrangements” present barriers to achieving tenure security and equitable distribution of NCBs, discouraging farmers from engaging in on-farm tree planting (Saeed et al. 2018, p. 48).

### Methodological Contribution and Research Limitations

This study employed an exploratory methodological approach, combining Q-methodology with semi-structured interviews and focus group discussions to identify four main narratives on NCBs from Ghana Cocoa REDD+. These methods complemented each other. While Q-methodology generated general perspectives, interviews with different actors in the cocoa sector generated contextual background information and added deeper insights into participants’ perceptions of and experiences with governance, social and economic NCBs. The focus group discussions allowed for group dynamics to obtain more nuanced and natural feedback. Additionally, the combination of methods allowed for a more diverse sample, thus providing a comprehensive range of perspectives from different actors involved in Ghana Cocoa REDD+.

Discrepancies arose between Q-methodology findings and the qualitative data from the other methods. For instance, while perspectives 2 and 3 from the Q-methodology suggested that the program should make seedlings readily available to farmers and that activities of intermediaries do not affect farmers’ incomes, the interviews and focus group discussions indicated that seedlings were not readily supplied, and activities of intermediaries did indeed affect farmers’ income. These differences align with the reliability concerns raised by van Exel and de Graaf (2005) and Thomas and Baas (1992) regarding Q-methodology.

Other limitations relate to the translation of the Q-statements into Twi, which may not have adequately conveyed the intended meaning due to challenges in finding appropriate wording for key terminologies. Additionally, the COVID-19 pandemic limited our access to representatives of the cocoa companies in particular. Despite these limitations, findings gathered from diverse actors through mixed methods provide valuable insights, though caution is advised in generalizing findings beyond this context.

## Conclusions

Q-methodology provided insights into four perspectives on the Hotspot Intervention Area (HIA) as a governance framework for generating governance, economic, and social non-carbon benefits (NCBs). The sorting and ranking of Q-statements also revealed the complexity and contested nature of community-led landscape governance frameworks, cocoa production, deforestation, livelihoods, and tree tenure. The combination of Q-methodology with semi-structured interviews and focus group discussions provided a richer and more contextualized understanding of landscape governance and the challenges standing in the way of building landscape resilience. This approach revealed several entry points for more inclusive and effective governance and sustainable cocoa production in a resilient Juabuso-Bia HIA.

We found renewed interest among actors in addressing cocoa-driven deforestation through the HIA but suggest greater dividends can be realized by addressing the weaknesses of CREMAs and piloting the HIA before a full-scale rollout. Forest communities identify more with the CREMAs than the HIA because of perceived ownership and better grounding in customary law and practices, leading to a higher likelihood of community support for CREMA-led initiatives. The HIA Management Board risks becoming a bureaucratic and centralized “command and control” governance unit, potentially overlooking the interests of farmers, women, and minority groups. These insights can be used to improve sub-national governance, enhance cross-sectoral collaboration, and promote environmental stewardship.

Recommendations for the Forestry Commission include revitalizing weak CREMAs and strengthening their leadership and democratic principles, enhancing community-level sensitization, building the capacity for collaborative governance of particularly women and vulnerable groups, ensuring coherence in governance instruments, disseminating information on the many instruments for effective landscape governance, and improve tree registration and tenure security. COCOBOD should improve the supply of farm inputs, foster climate-smart cocoa adoption, and pay realistic cocoa producer prices. Ideally, functional customary and statutory land and tree rights should be administered by the CREMAs to improve farmers’ tenure and income security.

Further studies on other HIAs within the Ghana Cocoa REDD+ program are recommended to assess their ability to reduce commodity-driven deforestation, provide social and economic benefits to cocoa communities, and ensure sustainable production of cocoa.

### Supplementary information

Supplementary Information

## Footnotes

1 Grievance Redress Mechanisms, HIA constitutions, the Juabuso-Bia framework of implementation of Ghana Cocoa REDD+ Program.

2 Although REDD+ focuses on forest governance, we also refer to landscape governance to acknowledge that forests are often part of a broader productive landscape.

3 The Ghana Cocoa Board is the state-controlled institution that regulates the cocoa trade and sets a minimum price for cocoa, decides which companies are licensed to buy cocoa from farmers, and provides support to the sector through extension services, pest and disease control programs, and quality control (https://cocobod.gh/objectives-of-board).

4 All appendices are in the supplementary material.

5 The first number references the statement, the second represent the ranking value of the statement and the third represents the z-score of the statement).
