# Supplementary material for: Actor Perceptions of the Governance Framework and Non-Carbon Benefits from the Ghana Cocoa Forest REDD+ Program: An Extended Q-Study of the Juabuso-Bia Hotspot Intervention Area
Source: Environ Manage. 2024 Apr 30;74(1):73–93. doi: 10.1007/s00267-024-01978-2 (PMC11208209; doi:10.1007/s00267-024-01978-2)
Supplement: Supplementary file 1 — Supplementary Information [file 267_2024_1978_MOESM1_ESM.docx]

**Supplementary Materials**

**Appendix 1 List of Q-Statements**

| **No.** | **Statement** |
| --- | --- |
| 1 | There is less promotion of organic cocoa compared to conventional/inorganic under the Ghana Cocoa Forest REDD+ Program. |
| 2 | The Ghana Cocoa Forest REDD+ Program will help communities to cope or adapt to the impacts of climate change. |
| 3 | Under the Ghana Cocoa Forest REDD+ Program, seedlings for climate-smart agriculture are readily and timeously provided. |
| 4 | The effect of climate change is influencing cocoa production negatively. |
| 5 | The Ghana Cocoa Forest REDD+ Program has led to a sustainable increase in cocoa production in the last 5 years. |
| 6 | The majority of farmers in the landscape adopt climate-smart agricultural practices on their farms. |
| 7 | As the focus of Ghana’s REDD+ in the high forest zone, cocoa presents a limited scope to address the challenge. |
| 8 | The Forestry Commission has the resources and capability to deliver on the Ghana Cocoa Forest REDD+ Program. |
| 9 | The Ghana Cocoa Forest REDD+ Program safeguards are adequate not to make CREMA communities worse off. |
| 10 | The Ghana Cocoa Forest REDD+ Program recognizes and involves communities in decision-making and project implementation. |
| 11 | There was extensive engagement with my community before and during Ghana Cocoa Forest REDD+ Program implementation. |
| 12 | I need a permit before picking non-timber forest products from the HIA/CREMA. |
| 13 | The main driver of deforestation and forest degradation in the high forest zone is cocoa production. |
| 14 | Natural resources in the HIA/CREMA are secured for the future due to Ghana Cocoa Forest REDD+ Program. |
| 15 | The aesthetic value of the landscape has improved since Ghana Cocoa Forest REDD+ Program. |
| 16 | Fuelwood harvesting and charcoal production have increased in the HIA/CREMA. |
| 17 | The quantity of non-timber forest products in the landscape has increased. |
| 18 | It is observed that rainfall volume has reduced, and patterns changed. |
| 19 | The Ghana Cocoa Forest REDD+ Program has affected recreation activities within the landscape. |
| 20 | There are increased activities of beneficial insects (pollinators) in support of cocoa production. |
| 21 | CREMA communities are becoming warmer due to increased temperature rise. |
| 22 | There is increased production of timber in the HIA/CREMA. |
| 23 | Forests in and around my community are getting smaller and are losing their value. |
| 24 | There is access to adequate freshwater for domestic purposes. |
| 25 | Commodity companies have not done much to address cocoa-driven deforestation. |
| 26 | I feel safe when accessing ecosystem services in the HIA/CREAM. |
| 27 | The Ghana Cocoa Forest REDD+ Program has reduced youth migration from the landscape to urban spaces for employment. |
| 28 | The Ghana Cocoa Forest REDD+ Program is an important intervention in reducing poverty among cocoa farmers. |
| 29 | The role of intermediaries in the sale of cocoa beans reduces farmers’ incomes. |
| 30 | Additional livelihood interventions of the Ghana Cocoa Forest REDD+ Program are adequate to provide a decent standard of living for HIA/CREMAs. |
| 31 | Women (cocoa) farmers’ incomes and economic well-being have improved under Ghana Cocoa Forest REDD+ Program. |
| 32 | The Living Income Differential (LID) is crucial for lifting cocoa farmers out of poverty. |
| 33 | Emission reduction will lead to improvement in livelihoods. |
| 34 | Enhanced livelihoods of cocoa farmers will lead to emission reductions in the landscape. |
| 35 | The Ghana Cocoa Forest REDD+ Program is a threat to livelihoods and employment opportunities in the CREMAs. |
| 36 | Steps were missed/ rushed during the development of governance structures to meet contract deadlines. |
| 37 | The Ghana Cocoa Forest REDD+ Program has adequate measures to address natural resource conflicts and community resistance. |
| 38 | Strengthened HIA/CREMA governance structures are one of the success stories of the Ghana Cocoa Forest REDD+ Program. |
| 39 | Politicians dominate national REDD+ policy discussions at the expense of experts. |
| 40 | The current climate change policy adequately addresses REDD+ implementation and practice. |
| 41 | The benefits I will accrue from the Ghana Cocoa Forest REDD+ Program are more than from my current land-use practices. |
| 42 | Carbon credit payments for Ghana Cocoa Forest REDD+ Program should go through national governments. |
| 43 | The Ghana Cocoa Forest REDD+ Program, framed around the CREMA concept, allows for the participation of communities and stakeholders. |
| 44 | There is effective collaboration among state agencies, the private sector, and civil society in the Ghana Cocoa Forest REDD+ Program. |
| 45 | I prefer individual cash benefits from performance-based carbon credit payments. |
| 46 | The current Ghana Cocoa Forest REDD+ Program’s benefit-sharing scheme has the potential to bring conflicts among stakeholders. |
| 47 | The Ghana Cocoa Forest REDD+ Program’s benefit-sharing scheme adequately addresses the exclusion of women and vulnerable groups. |
| 48 | Carbon credits secured by cocoa companies should be eligible for offsetting tax obligations. |
| 49 | The benefit-sharing scheme does not demonstrate fairness and equity for women and vulnerable groups. |
| 50 | Curbing deforestation through the Ghana Cocoa Forest REDD+ Program has compromised the developmental needs of the HIA/CREMA. |
| 51 | There is clarity on which institution leads the Ghana Cocoa Forest REDD+ Program implementation. |
| 52 | Within the last 5 years, the size of my cocoa farm in the HIA/CREMA has increased. |
| 53 | The Ghana Cocoa Forest REDD+ Program will lead to a (re) centralization of forest governance since the sales of carbon will be government-led. |
| 54 | Land/tree tenure regimes in the HIAs/CREMAs place women/vulnerable groups at the periphery of performance-based carbon credit payments. |
| 55 | Under the Ghana Cocoa Forest REDD+ Program, there are opportunities for women and migrants with no land-owning rights to benefit from carbon credits. |
| 56 | Weak tree rights and tenure are major drivers of tree loss in cocoa farms within the landscape. |
| 57 | The costs of tree rights registration will skew carbon payments to rich cocoa farmers to the detriment of poor ones. |
| 58 | Much has not been done to improve tree tenure security and registration for settlers/migrants and women in the Ghana Cocoa Forest REDD+ Program. |

**Appendix 2 Factor Loadings**

| **ID** | **Participant** | **Factor** | | | |
| --- | --- | --- | --- | --- | --- |
|  |  | **F1 –**  **Ghana Cocoa REDD+ Strengthens Sub-National Forest Governance** | **F2 – Prospects for Poverty Alleviation but Not for All** | **F3 – Effective Policy Design but Poor Stakeholder Collaboration and Selective Inclusion and Participation** | **F4 –**  **Land and Tree Tenure Rights and Resource Access are Not for the Poor** |
| G.1 | Government official | 0.09 | -0.03 | 0.08 | 0.71* |
| G.2 | Government official | 0.32* | 0.23 | 0.1 | 0.05 |
| G.3 | Government official | 0.28 | 0.3 | 0.41 | -0.24 |
| G.4 | Government official | 0.3 | -0.14 | -0.02 | 0.54* |
| N.1 | Non-governmental organization | 0.28 | -0.14 | -0.34 | 0.19 |
| N.2 | Non-governmental organization | 0.31* | -0.06 | 0.19 | -0.22 |
| N.3 | Non-governmental organization | 0.31 | -0.38 | -0.09 | -0.41 |
| N.4 | Non-governmental organization | 0.06 | -0.15 | -0.37* | 0.27 |
| R.1 | Researcher | 0.36 | -0.36 | -0.16 | 0.24 |
| R.2 | Researcher | -0.04 | -0.57* | -0.07 | 0.19 |
| CE.1 | HIA/CREMA executive | 0.53* | 0.45 | -0.08 | -0.05 |
| CE.2 | HIA/CREMA executive | 0.61* | -0.04 | -0.09 | 0.08 |
| CE 3 | HIA/CREMA executive | 0.41* | -0.11 | -0.03 | 0.04 |
| CE 4 | HIA/CREMA executive | 0.02 | 0.05 | 0.09 | -0.36* |
| W.1 | Female HIA/CREMA executive | 0.25 | 0.26 | 0.59* | -0.03 |
| W.2 | Female HIA/CREMA executive | 0.72* | 0.2 | 0.2 | -0.03 |
| PC.1 | Cocoa purchasing clerk | 0.56* | 0.07 | -0.04 | 0.2 |
| PC.2 | Cocoa purchasing clerk | 0.62* | 0.08 | -0.28 | 0.02 |
| F.1 | Farmer (Debiso) | 0.34 | 0.34 | -0.07 | -0.08 |
| F.2 | Farmer (Debiso) | 0.04 | -0.36* | -0.23 | 0.07 |
| F.3 | Farmer (Kwamekikrom) | 0.04 | 0.26* | 0.15 | -0.1 |
| F.4 | Farmer (Kwamekikrom) | 0.62* | 0.04 | 0.27 | -0.02 |
| F.5 | Farmer (Yawmantwa) | 0.01 | 0.49* | -0.04 | 0.44 |
| F.6 | Farmer (Asuopri) | -0.23 | 0.33* | 0.12 | -0.09 |
| F.7 | Farmer (Essam) | 0.62* | 0.13 | 0.25 | 0 |
| F.8 | Farmer (Kafas) | 0.07 | -0.19 | 0.82* | 0.2 |
| F.9 | Farmer (Nseim) | 0.03 | -0.09 | 0.14 | 0.57* |
| F.10 | Farmer (Nseim) | 0.28 | 0.66* | -0.25 | 0.16 |
| F.11 | Farmer (Juabuso sub-HIA) | -0.03 | -0.11 | 0.76* | 0.25 |
| FL.1 | Farm Laborer (Bia-sub-HIA) | 0.28 | 0.63* | -0.25 | 0.06 |

Note: * significance level: p-value <0 *.*05.

**Appendix 3 Plot of Statements Based on Z-score of Participants’ Perceptions on HIA Facilitating the Generation of Governance and Economic NCBs**


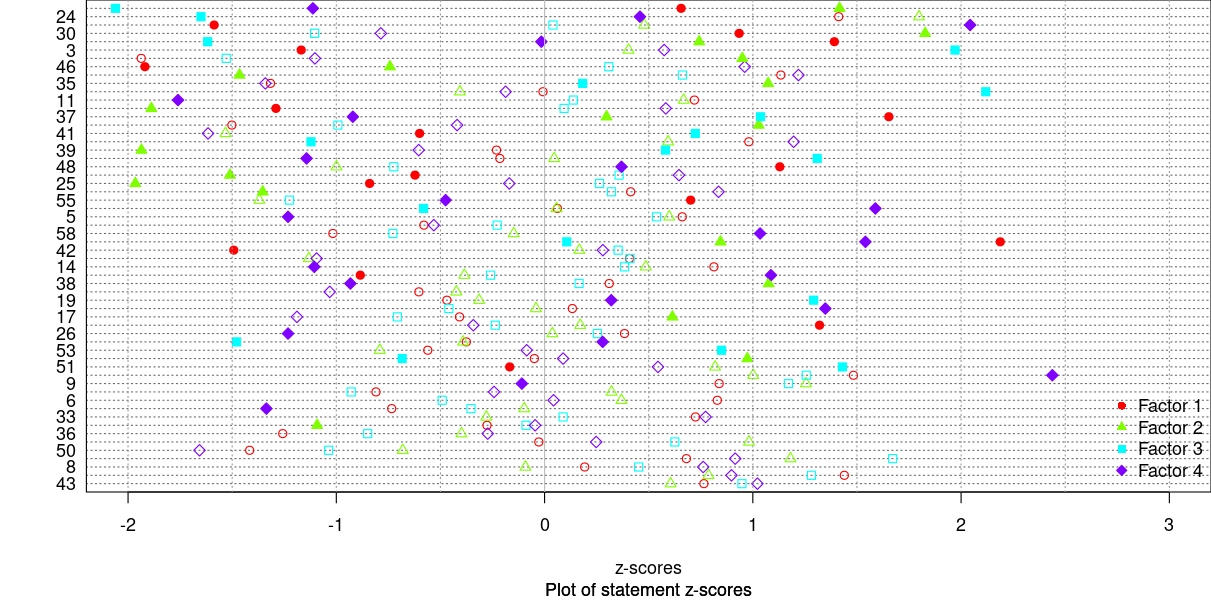


| **No** | **Statement** |
| --- | --- |
| 24 | There is access to adequate freshwater for domestic purposes. |
| 30 | Additional livelihood interventions of the Ghana Cocoa Forest REDD+ Program are adequate to provide a decent standard of living for HIA/CREMAs. |
| 3 | Under the Ghana Cocoa Forest REDD+ Program, seedlings for climate-smart agriculture are readily provided. |
| 46 | The current Ghana Cocoa Forest REDD+ Program’s benefit-sharing scheme has the potential to bring conflicts among stakeholders. |
| 35 | The Ghana Cocoa Forest REDD+ Program is a threat to livelihoods and employment opportunities in the CREMAs. |
| 11 | There was extensive engagement with my community before and during Ghana Cocoa Forest REDD+. |
| 37 | The Ghana Cocoa Forest REDD+ Program has adequate measures to address natural resource conflicts and community resistance. |
| 41 | The benefits I will accrue from the Ghana Cocoa Forest REDD+ Program are more than my current land-use practices. |
| 39 | Politicians dominate national REDD+ policy discussions at the expense of experts. |
| 48 | Carbon credits secured by cocoa companies should be eligible for offsetting tax obligations. |
| 25 | Commodity companies have not done much to address cocoa-driven deforestation. |
| 55 | Under the Ghana Cocoa Forest REDD+ Program, there are opportunities for women and migrants with no land-owning rights to benefit from carbon credits. |
| 5 | The Ghana Cocoa Forest REDD+ Program has led to an increase in cocoa production in the last 5 years. |
| 58 | Much has not been done to improve tree tenure security and registration for settlers/migrants and women in the Ghana Cocoa Forest REDD+ Program. |
| 42 | Carbon credit payments for Ghana Cocoa Forest REDD+ Program should go through national governments. |
| 14 | Natural resources in the HIA/CREMA are secured for the future due to Ghana Cocoa Forest REDD+ Program. |
| 38 | Strengthened HIA/CREMA governance is one of the success stories of the Ghana Cocoa Forest REDD+ Program. |
| 19 | The Ghana Cocoa Forest REDD+ Program has affected recreation activities within the landscape. |
| 17 | The quantity of non-timber forest products in the landscape has increased. |
| 26 | I feel safe when accessing ecosystem services in the HIA/CREAM. |
| 53 | The Ghana Cocoa Forest REDD+ Program will lead to a (re) centralization of forest governance since the sales of carbon will be government-led. |
| 51 | There is clarity on which institution leads the Ghana Cocoa Forest REDD+ Program implementation. |
| 6 | The majority of farmers in the landscape adopt climate-smart agricultural practices on their farms. |
| 9 | The Ghana Cocoa Forest REDD+ Program safeguards are adequate not to make communities worse off. |
| 33 | Emission reduction will lead to improvement in livelihoods. |
| 36 | Steps were missed/ rushed during the development of governance structures to meet contract deadlines. |
| 50 | Curbing deforestation through the Ghana Cocoa Forest REDD+ Program has compromised the developmental needs of the HIA/CREMA. |
| 8 | The Forestry Commission has the resources and capability to deliver on the Ghana Cocoa Forest REDD+. |
| 43 | The Ghana Cocoa Forest REDD+ Program, framed around the CREMA concept, allows for the participation of communities and stakeholders. |

**Missing Statements**

Some of the statements were not shown on the plot because, typically, the plot displays statements that show the most extreme scores. The focus is usually to showcase the statements that exhibit the strongest or most distinct viewpoints to facilitate interpretation and understanding of the different perspectives within the sample. Statements with higher or lower z-scores that exhibit more variability among participants were prioritized for display. Some statements may receive similar rankings across participants, resulting in z-scores that are close to zero. Such statements might not be visually represented on the plot as distinct points separate from the origin. It is also probable that the number of statements did not make it feasible to include all of them in the plot due to space limitations. (Zabala 2014; van Exel & de Graaf 2005; Watts, & Stenner 2012)

**Appendix 4 General Factor Characteristics**

| **Factor Coefficient Loading Eigenvalues Variance Reliability Factor** **scores** |
| --- |
| Factor 1 0.8 10 3.88 12.94 0.98 0.16 |
| Factor 2 0.8 7 2.70 9.01 0.97 0.19 |
| Factor 3 0.8 4 2.60 8.68 0.94 0.24 |
| Factor 4 0.8 4 2.14 7.14 0.94 0.24 |
|  |

The selection of perspectives was based on significant loading of two or more sorts in a factor and eigenvalues of 1.00 or higher as shown above.

**Appendix 5 Perspective 1 – The Ghana REDD+ Program Strengthens Sub-National Forest Governance Structures**

| **Statement number** | **Statement** | **Ranking value** | **z-score** |
| --- | --- | --- | --- |
| 1 | There is less promotion of organic cocoa compared to conventional/inorganic under the Ghana Cocoa Forest REDD+ Program. | -3 | -1.5 |
| 2 | The Ghana Cocoa Forest REDD+ Program will help communities to cope or adapt to the impacts of climate change. | 4 | 2.19 |
| 3 | Under the Ghana Cocoa Forest REDD+ Program, seedlings for climate-smart agriculture, are readily and timeously provided. | -2 | -1.17 |
| 4 | The effect of climate change is influencing cocoa production negatively. | 4 | 1.48 |
| 5 | The Ghana Cocoa Forest REDD+ Program has led to a sustainable increase in cocoa production in the last 5 years. | 1 | 0.66 |
| 6 | The majority of farmers in the landscape adopt climate- smart agricultural practices on their farms. | 2 | 0.83 |
| 7 | As the focus of Ghana’s REDD+ in the high forest zone, cocoa presents a limited scope to address the challenge. | -2 | -0.81 |
| 8 | The Forestry Commission has the resources and capability to deliver on the Ghana Cocoa Forest REDD+ Program. | 0 | 0.19 |
| 9 | The Ghana Cocoa Forest REDD+ Program safeguards are adequate not to make CREMA communities worse off. | 2 | 0.84 |
| 10 | The Ghana Cocoa Forest REDD+ Program recognizes and involves communities in decision-making and project implementation. | 3 | 1.39 |
| 11 | There was extensive engagement with my community before and during Ghana Cocoa Forest REDD+ Program implementation. | 1 | 0.72 |
| 12 | I need a permit before picking non-timber forest products from the HIA/CREMA. | 2 | 0.98 |
| 13 | The main driver of deforestation and forest degradation in the high forest zone is cocoa production. | 1 | 0.41 |
| 14 | Natural resources in the HIA/CREMA are secured for the future due to Ghana Cocoa Forest REDD+ Program. | 2 | 0.81 |
| 15 | The aesthetic value of the landscape has improved since Ghana Cocoa Forest REDD+ Program. | 0 | -0.03 |
| 16 | Fuelwood harvesting and charcoal production have increased in the HIA/CREMA. | -4 | -1.94 |
| 17 | The quantity of non-timber forest products in the landscape has increased. | -1 | -0.41 |
| 18 | It is observed that rainfall volume has reduced, and patterns changed. | 1 | 0.41 |
| 19 | The Ghana Cocoa Forest REDD+ Program has affected recreation activities within the landscape. | -1 | -0.47 |
| 20 | There are increased activities of beneficial insects (pollinators) in support of cocoa production. | 1 | 0.66 |
| 21 | CREMA communities are becoming warmer due to increased temperature rise. | -1 | -0.28 |
| 22 | There is increased production of timber in the HIA/CREMA. | -4 | -1.59 |
| 23 | Forests in and around my community are getting smaller and are losing their value. | 3 | 1.13 |
| 24 | There is access to adequate freshwater for domestic purposes. | 3 | 1.41 |
| 25 | Commodity companies have not done much to address cocoa-driven deforestation. | -2 | -0.84 |
| 26 | I feel safe when accessing ecosystem services in the HIA/CREAM. | 1 | 0.38 |
| 27 | The Ghana Cocoa Forest REDD+ Program has reduced youth migration from the landscape to urban spaces for employment. | -2 | -0.73 |
| 28 | The Ghana Cocoa Forest REDD+ Program is an important intervention in reducing poverty among cocoa farmers. | 1 | 0.68 |
| 29 | The role of intermediaries in the sale of cocoa beans reduces farmers’ incomes. | -1 | -0.58 |
| 30 | Additional livelihood interventions of the Ghana Cocoa Forest REDD+ Program are adequate to provide a decent standard of living for HIA/CREMAs. | 2 | 0.93 |
| 31 | Women (cocoa) farmers’ incomes and economic well-being have improved under Ghana Cocoa Forest REDD+ Program. | -2 | -0.88 |
| 32 | The Living Income Differential (LID) is crucial for lifting cocoa farmers out of poverty. | -1 | -0.38 |
| 33 | Emission reduction will lead to improvement in livelihoods. | 1 | 0.72 |
| 34 | Enhanced livelihoods of cocoa farmers will lead to emission reductions in the landscape. | 0 | -0.01 |
| 35 | The Ghana Cocoa Forest REDD+ Program is a threat to livelihoods and employment opportunities in the CREMAs. | -3 | -1.32 |
| 36 | Steps were missed/ rushed during the development of governance structures to meet contract deadlines. | -2 | -1.26 |
| 37 | The Ghana Cocoa Forest REDD+ Program has adequate measures to address natural resource conflicts and community resistance. | 4 | 1.65 |
| 38 | Strengthened HIA/CREMA governance structures are one of the success stories of the Ghana Cocoa Forest REDD+ Program. | 0 | 0.31 |
| 39 | Politicians dominate national REDD+ policy discussions at the expense of experts. | 0 | -0.23 |
| 40 | The current climate change policy adequately addresses REDD+ implementation and practice. | 3 | 1.44 |
| 41 | The benefits I will accrue from the Ghana Cocoa Forest REDD+ Program are more than from my current land-use practices. | -1 | -0.6 |
| 42 | Carbon credit payments for Ghana Cocoa Forest REDD+ Program should go through national governments. | -3 | -1.49 |
| 43 | The Ghana Cocoa Forest REDD+ Program, framed around the CREMA concept, allows for the participation of communities and stakeholders. | 2 | 0.77 |
| 44 | There is effective collaboration among state agencies, the private sector, and civil society in the Ghana Cocoa Forest REDD+ Program. | 3 | 1.32 |
| 45 | I prefer individual cash benefits from performance-based carbon credit payments. | 0 | 0.06 |
| 46 | The current Ghana Cocoa Forest REDD+ Program’s benefit-sharing scheme has the potential to bring conflicts among stakeholders. | -4 | -1.92 |
| 47 | The Ghana Cocoa Forest REDD+ Program’s benefit-sharing scheme adequately addresses the exclusion of women and vulnerable groups. | -3 | -1.29 |
| 48 | Carbon credits secured by cocoa companies should be eligible for offsetting tax obligations. | 2 | 1.13 |
| 49 | The benefit-sharing scheme does not demonstrate fairness and equity for women and vulnerable groups. | 0 | -0.05 |
| 50 | Curbing deforestation through the Ghana Cocoa Forest REDD+ Program has compromised the developmental needs of the HIA/CREMA. | -3 | -1.42 |
| 51 | There is clarity on which institution leads the Ghana Cocoa Forest REDD+ Program implementation. | 0 | -0.17 |
| 52 | Within the last 5 years, the size of my cocoa farm in the HIA/CREMA has increased. | 0 | -0.21 |
| 53 | The Ghana Cocoa Forest REDD+ Program will lead to a (re) centralization of forest governance since the sales of carbon will be government-led. | -1 | -0.56 |
| 54 | Land/tree tenure regimes in the HIAs/CREMAs place women/vulnerable groups at the periphery of performance-based carbon credit payments. | -1 | -0.62 |
| 55 | Under the Ghana Cocoa Forest REDD+ Program, there are opportunities for women and migrants with no land-owning rights to benefit from carbon credits. | 1 | 0.7 |
| 56 | Weak tree rights and tenure are major drivers of tree loss in cocoa farms within the landscape. | 0 | 0.13 |
| 57 | The costs of tree rights registration will skew carbon payments to rich cocoa farmers to the detriment of poor ones. | -1 | -0.6 |
| 58 | Much has not been done to improve tree tenure security and registration for settlers/migrants and women in the Ghana Cocoa Forest REDD+ Program. | -2 | -1.02 |

**Appendix 6 Perspective 2 – Prospects for Poverty Alleviation but Not for All**

| **Statement number** | **Statement** | **Ranking**  **value** |  | **z-score** |
| --- | --- | --- | --- | --- |
| 1 | There is less promotion of organic cocoa compared to conventional/inorganic under the Ghana Cocoa Forest REDD+ Program. | 3 |  | 1.03 |
| 2 | The Ghana Cocoa Forest REDD+ Program will help communities to cope or adapt to the impacts of climate change. | 2 |  | 0.84 |
| 3 | Under the Ghana Cocoa Forest REDD+ Program, seedlings for climate-smart agriculture, are readily and timeously provided. | 1 |  | 0.4 |
| 4 | The effect of climate change is influencing cocoa production negatively. | 2 |  | 1 |
| 5 | The Ghana Cocoa Forest REDD+ Program has led to a sustainable increase in cocoa production in the last 5 years. | 1 |  | 0.6 |
| 6 | The majority of farmers in the landscape adopt climate- smart agricultural practices on their farms. | 0 |  | 0.37 |
| 7 | As the focus of Ghana’s REDD+ in the high forest zone, cocoa presents a limited scope to address the challenge. | 0 |  | 0.32 |
| 8 | The Forestry Commission has the resources and capability to deliver on the Ghana Cocoa Forest REDD+ Program. | 0 |  | -0.09 |
| 9 | The Ghana Cocoa Forest REDD+ Program safeguards are adequate not to make CREMA communities worse off. | 3 |  | 1.26 |
| 10 | The Ghana Cocoa Forest REDD+ Program recognizes and involves communities in decision-making and project implementation. | 1 |  | 0.74 |
| 11 | There was extensive engagement with my community before and during Ghana Cocoa Forest REDD+ Program implementation. | 1 |  | 0.67 |
| 12 | I need a permit before picking non-timber forest products from the HIA/CREMA. | 1 |  | 0.59 |
| 13 | The main driver of deforestation and forest degradation in the high forest zone is cocoa production. | -2 |  | -1.13 |
| 14 | Natural resources in the HIA/CREMA are secured for the future due to Ghana Cocoa Forest REDD+ Program. | 1 |  | 0.49 |
| 15 | The aesthetic value of the landscape has improved since Ghana Cocoa Forest REDD+ Program. | 2 |  | 0.98 |
| 16 | Fuelwood harvesting and charcoal production have increased in the HIA/CREMA. | 2 |  | 0.95 |
| 17 | The quantity of non-timber forest products in the landscape has increased. | 1 |  | 0.61 |
| 18 | It is observed that rainfall volume has reduced, and patterns changed. | -2 |  | -1.35 |
| 19 | The Ghana Cocoa Forest REDD+ Program has affected recreation activities within the landscape. | -1 |  | -0.31 |
| 20 | There are increased activities of beneficial insects (pollinators) in support of cocoa production. | 4 |  | 1.42 |
| 21 | CREMA communities are becoming warmer due to increased temperature rise. | -2 |  | -1.09 |
| 22 | There is increased production of timber in the HIA/CREMA. | 1 |  | 0.48 |
| 23 | Forests in and around my community are getting smaller and are losing their value. | -3 |  | -1.46 |
| 24 | There is access to adequate freshwater for domestic purposes. | 4 |  | 1.8 |
| 25 | Commodity companies have not done much to address cocoa-driven deforestation. | -4 |  | -1.96 |
| 26 | I feel safe when accessing ecosystem services in the HIA/CREAM. | 0 |  | 0.04 |
| 27 | The Ghana Cocoa Forest REDD+ Program has reduced youth migration from the landscape to urban spaces for employment. | -1 |  | -0.1 |
| 28 | The Ghana Cocoa Forest REDD+ Program is an important intervention in reducing poverty among cocoa farmers. | 3 |  | 1.18 |
| 29 | The role of intermediaries in the sale of cocoa beans reduces farmers’ incomes. | -4 |  | -2.24 |
| 30 | Additional livelihood interventions of the Ghana Cocoa Forest REDD+ Program are adequate to provide a decent standard of living for HIA/CREMAs. | 4 |  | 1.83 |
| 31 | Women (cocoa) farmers’ incomes and economic well-being have improved under Ghana Cocoa Forest REDD+ Program. | -1 |  | -0.39 |
| 32 | The Living Income Differential (LID) is crucial for lifting cocoa farmers out of poverty. | -1 |  | -0.39 |
| 33 | Emission reduction will lead to improvement in livelihoods. | -1 |  | -0.28 |
| 34 | Enhanced livelihoods of cocoa farmers will lead to emission reductions in the landscape. | -1 |  | -0.41 |
| 35 | The Ghana Cocoa Forest REDD+ Program is a threat to livelihoods and employment opportunities in the CREMAs. | 3 |  | 1.07 |
| 36 | Steps were missed/ rushed during the development of governance structures to meet contract deadlines. | -1 |  | -0.4 |
| 37 | The Ghana Cocoa Forest REDD+ Program has adequate measures to address natural resource conflicts and community resistance. | 0 |  | 0.3 |
| 38 | Strengthened HIA/CREMA governance structures are one of the success stories of the Ghana Cocoa Forest REDD+ Program. | 3 |  | 1.08 |
| 39 | Politicians dominate national REDD+ policy discussions at the expense of experts. | -4 |  | -1.94 |
| 40 | The current climate change policy adequately addresses REDD+ implementation and practice. | 2 |  | 0.79 |
| 41 | The benefits I will accrue from the Ghana Cocoa Forest REDD+ Program are more than from my current land-use practices. | -3 |  | -1.53 |
| 42 | Carbon credit payments for Ghana Cocoa Forest REDD+ Program should go through national governments. | 0 |  | 0.17 |
| 43 | The Ghana Cocoa Forest REDD+ Program, framed around the CREMA concept, allows for the participation of communities and stakeholders. | 1 |  | 0.61 |
| 44 | There is effective collaboration among state agencies, the private sector, and civil society in the Ghana Cocoa Forest REDD+ Program. | 0 |  | 0.17 |
| 45 | I prefer individual cash benefits from performance-based carbon credit payments. | 0 |  | 0.06 |
| 46 | The current Ghana Cocoa Forest REDD+ Program’s benefit-sharing scheme has the potential to bring conflicts among stakeholders. | -2 |  | -0.74 |
| 47 | The Ghana Cocoa Forest REDD+ Program’s benefit-sharing scheme adequately addresses the exclusion of women and vulnerable groups. | -3 |  | -1.89 |
| 48 | Carbon credits secured by cocoa companies should be eligible for offsetting tax obligations. | -2 |  | -1 |
| 49 | The benefit-sharing scheme does not demonstrate fairness and equity for women and vulnerable groups. | 2 |  | 0.97 |
| 50 | Curbing deforestation through the Ghana Cocoa Forest REDD+ Program has compromised the developmental needs of the HIA/CREMA. | -2 |  | -0.68 |
| 51 | There is clarity on which institution leads the Ghana Cocoa Forest REDD+ Program implementation. | 2 |  | 0.82 |
| 52 | Within the last 5 years, the size of my cocoa farm in the HIA/CREMA has increased. | 0 |  | 0.05 |
| 53 | The Ghana Cocoa Forest REDD+ Program will lead to a (re) centralization of forest governance since the sales of carbon will be government-led. | -2 |  | -0.79 |
| 54 | Land/tree tenure regimes in the HIAs/CREMAs place women/vulnerable groups at the periphery of performance-based carbon credit payments. | -3 |  | -1.51 |
| 55 | Under the Ghana Cocoa Forest REDD+ Program, there are opportunities for women and migrants with no land-owning rights to benefit from carbon credits. | -3 |  | -1.37 |
| 56 | Weak tree rights and tenure are major drivers of tree loss in cocoa farms within the landscape. | 0 |  | -0.04 |
| 57 | The costs of tree rights registration will skew carbon payments to rich cocoa farmers to the detriment of poor ones. | -1 |  | -0.42 |
| 58 | Much has not been done to improve tree tenure security and registration for settlers/migrants and women in the Ghana Cocoa Forest REDD+ Program. | -1 |  | -0.15 |

**Appendix 7** Perspective 3 **– Effective Policy Design but Poor Stakeholder Collaboration and Selective Inclusion and Participation**

| **Statement number** | **Statement** | **Ranking**  **value** | **z-score** |
| --- | --- | --- | --- |
| 1 | There is less promotion of organic cocoa compared to conventional/inorganic under the Ghana Cocoa Forest REDD+ Program. | -2 | -0.99 |
| 2 | The Ghana Cocoa Forest REDD+ Program will help communities to cope or adapt to the impacts of climate change. | 0 | 0.11 |
| 3 | Under the Ghana Cocoa Forest REDD+ Program, seedlings for climate-smart agriculture, are readily and timeously provided. | 4 | 1.97 |
| 4 | The effect of climate change is influencing cocoa production negatively. | 3 | 1.26 |
| 5 | The Ghana Cocoa Forest REDD+ Program has led to a sustainable increase in cocoa production in the last 5 years. | 1 | 0.54 |
| 6 | The majority of farmers in the landscape adopt climate- smart agricultural practices on their farms. | -1 | -0.49 |
| 7 | As the focus of Ghana’s REDD+ in the high forest zone, cocoa presents a limited scope to address the challenge. | -2 | -0.93 |
| 8 | The Forestry Commission has the resources and capability to deliver on the Ghana Cocoa Forest REDD+ Program. | 1 | 0.45 |
| 9 | The Ghana Cocoa Forest REDD+ Program safeguards are adequate not to make CREMA communities worse off. | 2 | 1.17 |
| 10 | The Ghana Cocoa Forest REDD+ Program recognizes and involves communities in decision-making and project implementation. | -3 | -1.62 |
| 11 | There was extensive engagement with my community before and during Ghana Cocoa Forest REDD+ Program implementation. | 0 | 0.14 |
| 12 | I need a permit before picking non-timber forest products from the HIA/CREMA. | -3 | -1.12 |
| 13 | The main driver of deforestation and forest degradation in the high forest zone is cocoa production. | 1 | 0.41 |
| 14 | Natural resources in the HIA/CREMA are secured for the future due to Ghana Cocoa Forest REDD+ Program. | 1 | 0.38 |
| 15 | The aesthetic value of the landscape has improved since Ghana Cocoa Forest REDD+ Program. | 2 | 0.63 |
| 16 | Fuelwood harvesting and charcoal production have increased in the HIA/CREMA. | -3 | -1.53 |
| 17 | The quantity of non-timber forest products in the landscape has increased. | -1 | -0.71 |
| 18 | It is observed that rainfall volume has reduced, and patterns changed. | 1 | 0.32 |
| 19 | The Ghana Cocoa Forest REDD+ Program has affected recreation activities within the landscape. | 3 | 1.29 |
| 20 | There are increased activities of beneficial insects (pollinators) in support of cocoa production. | -4 | -2.06 |
| 21 | CREMA communities are becoming warmer due to increased temperature rise. | 0 | -0.09 |
| 22 | There is increased production of timber in the HIA/CREMA. | 0 | 0.04 |
| 23 | Forests in and around my community are getting smaller and are losing their value. | 2 | 0.66 |
| 24 | There is access to adequate freshwater for domestic purposes. | -4 | -1.65 |
| 25 | Commodity companies have not done much to address cocoa-driven deforestation. | 0 | 0.26 |
| 26 | I feel safe when accessing ecosystem services in the HIA/CREAM. | 0 | 0.25 |
| 27 | The Ghana Cocoa Forest REDD+ Program has reduced youth migration from the landscape to urban spaces for employment. | -1 | -0.35 |
| 28 | The Ghana Cocoa Forest REDD+ Program is an important intervention in reducing poverty among cocoa farmers. | 4 | 1.67 |
| 29 | The role of intermediaries in the sale of cocoa beans reduces farmers’ incomes. | -1 | -0.23 |
| 30 | Additional livelihood interventions of the Ghana Cocoa Forest REDD+ Program are adequate to provide a decent standard of living for HIA/CREMAs. | -2 | -1.1 |
| 31 | Women (cocoa) farmers’ incomes and economic well-being have improved under Ghana Cocoa Forest REDD+ Program. | -1 | -0.26 |
| 32 | The Living Income Differential (LID) is crucial for lifting cocoa farmers out of poverty. | -3 | -1.48 |
| 33 | Emission reduction will lead to improvement in livelihoods. | 0 | 0.09 |
| 34 | Enhanced livelihoods of cocoa farmers will lead to emission reductions in the landscape. | 4 | 2.12 |
| 35 | The Ghana Cocoa Forest REDD+ Program is a threat to livelihoods and employment opportunities in the CREMAs. | 0 | 0.18 |
| 36 | Steps were missed/ rushed during the development of governance structures to meet contract deadlines. | -2 | -0.85 |
| 37 | The Ghana Cocoa Forest REDD+ Program has adequate measures to address natural resource conflicts and community resistance. | 2 | 1.04 |
| 38 | Strengthened HIA/CREMA governance structures are one of the success stories of the Ghana Cocoa Forest REDD+ Program. | 0 | 0.17 |
| 39 | Politicians dominate national REDD+ policy discussions at the expense of experts. | 1 | 0.58 |
| 40 | The current climate change policy adequately addresses REDD+ implementation and practice. | 3 | 1.28 |
| 41 | The benefits I will accrue from the Ghana Cocoa Forest REDD+ Program are more than from my current land-use practices. | 2 | 0.72 |
| 42 | Carbon credit payments for Ghana Cocoa Forest REDD+ Program should go through national governments. | 1 | 0.35 |
| 43 | The Ghana Cocoa Forest REDD+ Program, framed around the CREMA concept, allows for the participation of communities and stakeholders. | 2 | 0.95 |
| 44 | There is effective collaboration among state agencies, the private sector, and civil society in the Ghana Cocoa Forest REDD+ Program. | -1 | -0.24 |
| 45 | I prefer individual cash benefits from performance-based carbon credit payments. | -1 | -0.58 |
| 46 | The current Ghana Cocoa Forest REDD+ Program’s benefit-sharing scheme has the potential to bring conflicts among stakeholders. | 1 | 0.31 |
| 47 | The Ghana Cocoa Forest REDD+ Program’s benefit-sharing scheme adequately addresses the exclusion of women and vulnerable groups. | 0 | 0.09 |
| 48 | Carbon credits secured by cocoa companies should be eligible for offsetting tax obligations. | -2 | -0.72 |
| 49 | The benefit-sharing scheme does not demonstrate fairness and equity for women and vulnerable groups. | -1 | -0.68 |
| 50 | Curbing deforestation through the Ghana Cocoa Forest REDD+ Program has compromised the developmental needs of the HIA/CREMA. | -2 | -1.04 |
| 51 | There is clarity on which institution leads the Ghana Cocoa Forest REDD+ Program implementation. | 3 | 1.43 |
| 52 | Within the last 5 years, the size of my cocoa farm in the HIA/CREMA has increased. | 3 | 1.31 |
| 53 | The Ghana Cocoa Forest REDD+ Program will lead to a (re) centralization of forest governance since the sales of carbon will be government-led. | 2 | 0.85 |
| 54 | Land/tree tenure regimes in the HIAs/CREMAs place women/vulnerable groups at the periphery of performance-based carbon credit payments. | 1 | 0.36 |
| 55 | Under the Ghana Cocoa Forest REDD+ Program, there are opportunities for women and migrants with no land-owning rights to benefit from carbon credits. | -3 | -1.22 |
| 56 | Weak tree rights and tenure are major drivers of tree loss in cocoa farms within the landscape. | -1 | -0.46 |
| 57 | The costs of tree rights registration will skew carbon payments to rich cocoa farmers to the detriment of poor ones. | -4 | -2.25 |
| 58 | Much has not been done to improve tree tenure security and registration for settlers/migrants and women in the Ghana Cocoa Forest REDD+ Program. | -2 | -0.73 |

**Appendix 8 Perspective 4 – Land and Tree Tenure Rights and Natural Resource Access Are Not for the Poor**

| Statement Number | Statement | Ranking value | z-score |
| --- | --- | --- | --- |
| 1 | There is less promotion of organic cocoa compared to conventional/inorganic under the Ghana Cocoa Forest REDD+ Program. | -1 | -0.42 |
| 2 | The Ghana Cocoa Forest REDD+ Program will help communities to cope or adapt to the impacts of climate change. | 3 | 1.54 |
| 3 | Under the Ghana Cocoa Forest REDD+ Program, seedlings for climate-smart agriculture, are readily and timeously provided. | 1 | 0.57 |
| 4 | The effect of climate change is influencing cocoa production negatively. | 4 | 2.44 |
| 5 | The Ghana Cocoa Forest REDD+ Program has led to a sustainable increase in cocoa production in the last 5 years. | -3 | -1.23 |
| 6 | The majority of farmers in the landscape adopt climate- smart agricultural practices on their farms. | 0 | 0.04 |
| 7 | As the focus of Ghana’s REDD+ in the high forest zone, cocoa presents a limited scope to address the challenge. | -1 | -0.24 |
| 8 | The Forestry Commission has the resources and capability to deliver on the Ghana Cocoa Forest REDD+ Program. | 1 | 0.76 |
| 9 | The Ghana Cocoa Forest REDD+ Program safeguards are adequate not to make CREMA communities worse off. | 0 | -0.11 |
| 10 | The Ghana Cocoa Forest REDD+ Program recognizes and involves communities in decision-making and project implementation. | 0 | -0.02 |
| 11 | There was extensive engagement with my community before and during Ghana Cocoa Forest REDD+ Program implementation. | -4 | -1.76 |
| 12 | I need a permit before picking non-timber forest products from the HIA/CREMA. | 3 | 1.2 |
| 13 | The main driver of deforestation and forest degradation in the high forest zone is cocoa production. | -2 | -1.09 |
| 14 | Natural resources in the HIA/CREMA are secured for the future due to Ghana Cocoa Forest REDD+ Program. | -2 | -1.11 |
| 15 | The aesthetic value of the landscape has improved since Ghana Cocoa Forest REDD+ Program. | 0 | 0.25 |
| 16 | Fuelwood harvesting and charcoal production have increased in the HIA/CREMA. | -2 | -1.1 |
| 17 | The quantity of non-timber forest products in the landscape has increased. | -3 | -1.19 |
| 18 | It is observed that rainfall volume has reduced, and patterns changed. | 2 | 0.84 |
| 19 | The Ghana Cocoa Forest REDD+ Program has affected recreation activities within the landscape. | 1 | 0.32 |
| 20 | There are increased activities of beneficial insects (pollinators) in support of cocoa production. | -2 | -1.11 |
| 21 | CREMA communities are becoming warmer due to increased temperature rise. | 0 | -0.05 |
| 22 | There is increased production of timber in the HIA/CREMA. | 4 | 2.04 |
| 23 | Forests in and around my community are getting smaller and are losing their value. | 3 | 1.22 |
| 24 | There is access to adequate freshwater for domestic purposes. | 1 | 0.46 |
| 25 | Commodity companies have not done much to address cocoa-driven deforestation. | 0 | -0.17 |
| 26 | I feel safe when accessing ecosystem services in the HIA/CREAM. | -3 | -1.23 |
| 27 | The Ghana Cocoa Forest REDD+ Program has reduced youth migration from the landscape to urban spaces for employment. | -3 | -1.34 |
| 28 | The Ghana Cocoa Forest REDD+ Program is an important intervention in reducing poverty among cocoa farmers. | 2 | 0.92 |
| 29 | The role of intermediaries in the sale of cocoa beans reduces farmers’ incomes. | -1 | -0.53 |
| 30 | Additional livelihood interventions of the Ghana Cocoa Forest REDD+ Program are adequate to provide a decent standard of living for HIA/CREMAs. | -1 | -0.79 |
| 31 | Women (cocoa) farmers’ incomes and economic well-being have improved under Ghana Cocoa Forest REDD+ Program. | 3 | 1.09 |
| 32 | The Living Income Differential (LID) is crucial for lifting cocoa farmers out of poverty. | 0 | 0.28 |
| 33 | Emission reduction will lead to improvement in livelihoods. | 2 | 0.77 |
| 34 | Enhanced livelihoods of cocoa farmers will lead to emission reductions in the landscape. | 0 | -0.19 |
| 35 | The Ghana Cocoa Forest REDD+ Program is a threat to livelihoods and employment opportunities in the CREMAs. | -3 | -1.34 |
| 36 | Steps were missed/ rushed during the development of governance structures to meet contract deadlines. | -1 | -0.27 |
| 37 | The Ghana Cocoa Forest REDD+ Program has adequate measures to address natural resource conflicts and community resistance. | -1 | -0.92 |
| 38 | Strengthened HIA/CREMA governance structures are one of the success stories of the Ghana Cocoa Forest REDD+ Program. | -2 | -0.93 |
| 39 | Politicians dominate national REDD+ policy discussions at the expense of experts. | -1 | -0.6 |
| 40 | The current climate change policy adequately addresses REDD+ implementation and practice. | 2 | 0.9 |
| 41 | The benefits I will accrue from the Ghana Cocoa Forest REDD+ Program are more than from my current land-use practices. | -4 | -1.62 |
| 42 | Carbon credit payments for Ghana Cocoa Forest REDD+ Program should go through national governments. | 1 | 0.28 |
| 43 | The Ghana Cocoa Forest REDD+ Program, framed around the CREMA concept, allows for the participation of communities and stakeholders. | 2 | 1.02 |
| 44 | There is effective collaboration among state agencies, the private sector, and civil society in the Ghana Cocoa Forest REDD+ Program. | -1 | -0.34 |
| 45 | I prefer individual cash benefits from performance-based carbon credit payments. | 4 | 1.59 |
| 46 | The current Ghana Cocoa Forest REDD+ Program’s benefit-sharing scheme has the potential to bring conflicts among stakeholders. | 2 | 0.96 |
| 47 | The Ghana Cocoa Forest REDD+ Program’s benefit-sharing scheme adequately addresses the exclusion of women and vulnerable groups. | 1 | 0.58 |
| 48 | Carbon credits secured by cocoa companies should be eligible for offsetting tax obligations. | 1 | 0.37 |
| 49 | The benefit-sharing scheme does not demonstrate fairness and equity for women and vulnerable groups. | 0 | 0.09 |
| 50 | Curbing deforestation through the Ghana Cocoa Forest REDD+ Program has compromised the developmental needs of the HIA/CREMA. | -4 | -1.66 |
| 51 | There is clarity on which institution leads the Ghana Cocoa Forest REDD+ Program implementation. | 1 | 0.54 |
| 52 | Within the last 5 years, the size of my cocoa farm in the HIA/CREMA has increased. | -2 | -1.14 |
| 53 | The Ghana Cocoa Forest REDD+ Program will lead to a (re) centralization of forest governance since the sales of carbon will be government-led. | 0 | -0.09 |
| 54 | Land/tree tenure regimes in the HIAs/CREMAs place women/vulnerable groups at the periphery of performance-based carbon credit payments. | 1 | 0.65 |
| 55 | Under the Ghana Cocoa Forest REDD+ Program, there are opportunities for women and migrants with no land-owning rights to benefit from carbon credits. | -1 | -0.48 |
| 56 | Weak tree rights and tenure are major drivers of tree loss in cocoa farms within the landscape. | 3 | 1.35 |
| 57 | The costs of tree rights registration will skew carbon payments to rich cocoa farmers to the detriment of poor ones. | -2 | -1.03 |
| 58 | Much has not been done to improve tree tenure security and registration for settlers/migrants and women in the Ghana Cocoa Forest REDD+ Program. | 2 | 1.03 |

**Appendix 9 Distinguishing** **and Consensus Statements from the Q-sort Analysis**

Distinguishing statements

| **Statement**  **Number** | **Statement** | **Ranking per factor** | | | |
| --- | --- | --- | --- | --- | --- |
|  |  | **F1** | **F2** | **F3** | **F4** |
| 2 | The Ghana Cocoa Forest REDD+ Program will help communities to cope or adapt to the impacts of climate change. | 4 | 2 | 0 | 3 |
| 10 | The Ghana Cocoa Forest REDD+ Program recognizes and involves communities in decision-making and project implementation. | 3 | 1 | -3 | 0 |
| 20 | There are increased activities of beneficial insects (pollinators) in support of cocoa production. | 1 | 4 | -4 | -2 |
| 37 | The Ghana Cocoa Forest REDD+ Program has adequate measures to address natural resource conflicts and community resistance. | 4 | 0 | 2 | -1 |

Consensus statement

| **Number** | **Statement** | **Ranking per factor** | | | |
| --- | --- | --- | --- | --- | --- |
|  |  | **F1** | **F2** | **F3** | **F4** |
| 43 | The Ghana Cocoa Forest REDD+ Program, framed around the CREMA concept, allows for the participation of communities and stakeholders. | 2 | 1 | 2 | 2 |
